# Advancing Heteroanionicity in Zintl Phases: Crystal Structures, Thermoelectric and Magnetic Properties of Two Quaternary Semiconducting Arsenide Oxides, Eu_8_Zn_2_As_6_O and Eu_14_Zn_5_As_12_O

**DOI:** 10.1021/acs.inorgchem.4c01580

**Published:** 2024-06-21

**Authors:** Mohd Ishtiyak, Spencer R. Watts, Bhushan Thipe, Frank Womack, Philip Adams, Xiaojian Bai, David P. Young, Svilen Bobev, Sviatoslav Baranets

**Affiliations:** 1 Department of Chemistry, Louisiana State University, Baton Rouge, Louisiana 70803, United States; 2 Department of Physics & Astronomy, Louisiana State University, Baton Rouge, Louisiana 70803, United States; 3 Department of Chemistry and Biochemistry, University of Delaware, Newark, Delaware 19716, United States

## Abstract

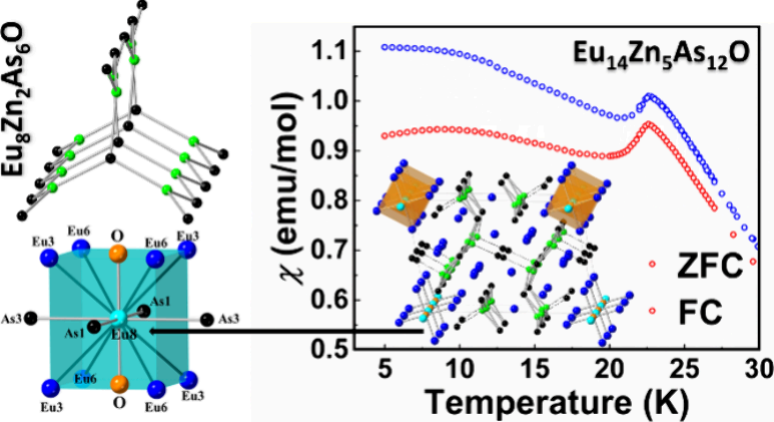

Two novel quaternary oxyarsenides, Eu_8_Zn_2_As_6_O and Eu_14_Zn_5_As_12_O, were synthesized through metal flux reactions, and their crystal structures were established by single-crystal X-ray diffraction methods. Eu_8_Zn_2_As_6_O crystallizes in the orthorhombic space group *Pbca*, featuring polyanionic ribbons composed of corner-shared triangular [ZnAs_3_] units, running along the [100] direction. The structure of Eu_14_Zn_5_As_12_O crystallizes in the monoclinic space group *P*2/*m* and its anionic substructure can be described as an infinite “ribbonlike” chain comprised of [ZnAs_3_] trigonal-planar units, although the structural complexity here is greater and also amplified by disorder on multiple crystallographic positions. In both structures, the O^2–^ anion occupies an octahedral void with six neighboring Eu^2+^ cations. Formal electron counting, electronic structure calculations, and transport properties reveal the charge-balanced semiconducting nature of these heteroanionic Zintl phases. High-temperature thermoelectric transport properties measurements on Eu_14_Zn_5_As_12_O reveal relatively high resistivity (*ρ*_500K_ = 8 Ω·cm) and Seebeck coefficient values (*S*_500K_ = 220 μV K^–1^), along with a low concentration and mobility of holes as the dominant charge-carriers (*n*_500K_ = 8.0 × 10^17^ cm^–3^, *μ*_500K_ = 6.4 cm^2^/V s). Magnetic studies indicate the presence of divalent Eu^2+^ species in Eu_14_Zn_5_As_12_O and complex magnetic ordering, with two transitions observed at *T*_1_ = 21.6 K and *T*_2_ = 9 K.

## Abstract 2

Two heteroanionic semiconducting oxyarsenides, Eu_8_Zn_2_As_6_O and Eu_14_Zn_5_As_12_O, adopt novel structure types. Their 1D anionic substructures are based on the corner-sharing arrangement of planar [ZnAs_3_] units surrounded by O-centered corner-sharing [Eu_6_] octahedra. Physical properties measured on single crystals of Eu_14_Zn_5_As_12_O reveal an intrinsic semiconducting behavior and complex magnetic behavior with two observed transitions at *T*_*N*__,1_ = 22.6(1) K and *T*_*N*__,2_ = 9.0(1) K.

## 1 Introduction

Pnictide oxides or oxypnictides belong to the class of heteroanionic inorganic materials characterized by the presence of oxide, O^2–^ and pnictide, *Pn*^3–^ (*Pn* = P, As, Sb, and Bi) anions with lack of direct *Pn*–O bonding.^1^ Their rich compositional and structural landscape originates from diverse coordination environments, sizes, and electronegativities of constituent anions, which enables the formation of compounds with unique structural motifs and remarkable physical properties, setting the stage for advancing existing and creating novel applications in technology and material science.^2^

Over the past decade, the exploration of novel heteroanionic oxypnictides has turned up many unique materials showing high-temperature superconductivity,^3,4^ interesting magnetism,^5−7^ topological properties,^8^ magnetoresistance,^9,10^ and potential for thermoelectric energy conversion.^11^ The 2008 discovery of high-temperature superconductivity in iron arsenide oxides significantly heightened interest in this class of compounds, albeit inadvertently overshadowing their tremendous potential as narrow band gap semiconductors.^4,12−18^

Notably, the structural chemistry of pnictide oxides often correlates with that of semiconducting Zintl pnictides, sharing similar compositional and structural traits, although the inclusion of oxide anions considerably expands the structural diversity and the range of potential applications.^19−21^ In many ways, the chemical bonding in oxypnictides fits within the framework of the Zintl–Klemm formalism,^22^ and the classic valence rules, with valence electron counts being “balanced” by considering electropositive metals acting as electron donors and the pnictogen and oxygen atoms as electron acceptors. As such, there is an expectation for observing narrow band gap semiconducting or semimetallic behavior in complex heteroanionic oxypnictides, although the presence of oxygen could introduce a greater degree of ionicity compared to the Zintl pnictides.

Our research groups have a longstanding interest in the development of novel Zintl materials for thermoelectric applications.^23,24^ Their small electronic band gaps, unique transport properties, along with their complex and often highly disordered structures contribute to their favorable thermoelectric performance. This performance can be quantified by the dimensionless figure of merit *zT* = *S*^2^*σT*/*κ*, where *σ* is the electrical conductivity, *S* is the Seebeck coefficient, *κ* is the thermal conductivity, and *T* is the absolute temperature.^25^

Within the realm of Zintl phases, pnictide compounds are among the most extensively investigated, particularly as leading p-type thermoelectric materials in high-temperature applications, with a *zT* > 1.^26−28^ However, the thermoelectric properties of closely related oxypnictides have been less explored, with only a few materials being studied, such as *RE*_2_SbO_2_ and *REMPn*O (*RE* = rare-earth element, *M* = Zn, Mn; *Pn* = As, Sb).^11,29−32^ The scant exploration into these materials has been largely due to the synthetic challenges of achieving phase-pure multinary compounds,^1^ although their complex crystal lattices, promising transport properties, and the features of their electronic structures identify these materials as promising candidates for thermoelectric applications, as recently predicted for the ternary semiconducting oxypnictides, Ca_4_*Pn*_2_O (*Pn* = Sb, Bi).^33^

**Figure 1:**
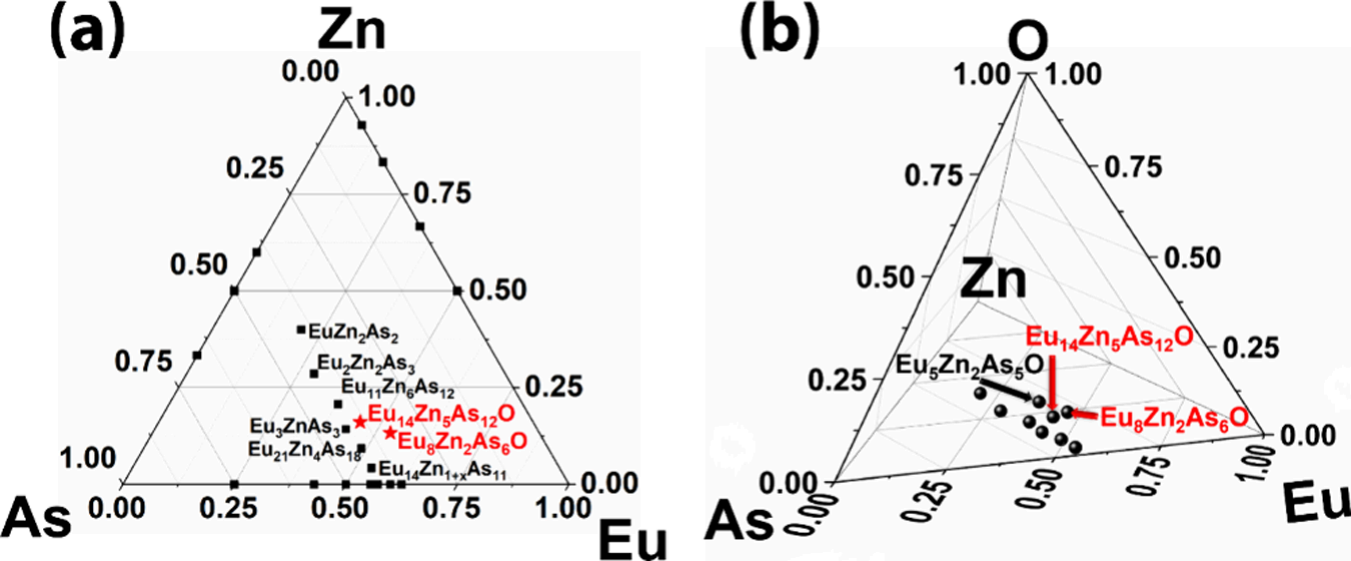
(a) Ternary Eu–Zn–As compositional diagram. The compositions of the newly identified quaternary Eu_8_Zn_2_As_6_O and Eu_14_Zn_5_As_12_O phases are projected on the plane and marked as red stars. (b) Quaternary Eu–Zn–As–O compositional diagram with new and reported ternary Eu–Zn–As and quaternary phases. Labels for the ternary compounds are omitted for clarity.

Inspired by the abundant structural and physical properties of the heteroanionic materials, we have discovered two novel quaternary oxypnictides, Eu_8_Zn_2_As_6_O and Eu_14_Zn_5_As_12_O. These compounds enrich the quaternary Eu–Zn–As–O compositional diagram (Figure 1b), which previously contained a single known compound, Eu_5_Zn_2_As_5_O (Ba_5_Cd_2_Sb_5_F structure type).^34^ In this study, we discuss synthetic challenges, offer a comprehensive structural analysis, and elucidate the electronic structure of these newly discovered complex, heavily disordered heteroanionic Zintl compounds. Our ability to synthesize large single crystals of Eu_14_Zn_5_As_12_O allowed for a comprehensive characterization of its physical properties. We report the study of the Seebeck coefficient, electrical resistivity, and Hall measurements, discuss the potential of this compound for thermoelectric applications, and conduct a preliminary study of its magnetic properties. We discuss structure–property relationships, linking extensive structural disorder with the theoretically predicted and experimentally validated semiconducting behavior.

## 2 Experimental Section

### 2.1 Synthesis

***Caution!** Arsenic and its compounds are hazardous. The synthesized phases may slowly hydrolyze, producing highly toxic arsane (AsH*_*3*_*), therefore, immediate cleaning of crucibles, tools, and labware with water is prohibited. Since the reaction temperature is significantly higher than the sublimation point of arsenic, all experimental procedures, including high-temperature treatment, must be performed in well-ventilated areas. Adequate personal protective equipment must always be used while working with synthesized compounds*.

The quaternary oxypnictides Eu_8_Zn_2_As_6_O and Eu_14_Zn_5_As_12_O were synthesized using the following chemicals as received from suppliers: Eu metal pieces (Edgetech Ind., 99.9%), Zn powder (J.T. Baker, 99.99%), As granules (ThermoFisher, 99.999%), and Pb powder (Strem Chemicals, 99.99%). Oxide layers on the europium metal pieces were removed using a metal file before cutting into smaller pieces. All handling was performed in an argon-filled glovebox with H_2_O and O_2_ levels maintained below 1 ppm or under vacuum conditions.

Both title compounds were synthesized employing a molten lead flux in a Eu-rich reaction mixture with loaded ratios in the range Eu:Zn:As:Pb ≈ 6–10:1:3–6:50, while maintaining the Eu:As ratio at ≈ 2:1 ratio. The synthesis process involved loading the reactants into an alumina crucible, which was then placed inside a fused silica tube. The crucible was covered with quartz wool, which served as a filter for the Pb-flux removal. The vessels were then flame-sealed under vacuum in a silica jacket and aligned vertically inside a muffle furnace. The reactants were heated to 1273 K at a rate of 100 K/h and annealed at this temperature for 24 h, followed by cooling to 923 K at a 5 K/h rate, removal from the furnace, and subsequent centrifugation at high speeds to discard the excess of Pb flux. After centrifugation, the ampoules were brought back to the glovebox and cracked open. Our findings indicate that Eu_8_Zn_2_As_6_O and Eu_14_Zn_5_As_12_O crystals are almost always obtained together, though reactions with lower (Eu/As):Zn ratios tend to favor the formation of the Eu_14_Zn_5_As_12_O phase, as can be suggested from the compositional diagram (Figure 1). Despite the absence of intentionally introduced oxygen, the title compounds are produced in high yields. This points to the presence of nonmetal impurities among the reactants, as discussed in greater detail in Section 3.1.

### 2.2 Single-Crystal X-ray Diffraction (SCXRD) Studies

The crystal structures of Eu_8_Zn_2_As_6_O and Eu_14_Zn_5_As_12_O oxypnictides were established using single crystal X-ray diffraction (SCXRD) technique on a Bruker D8 Venture DUO with a Photon III C14 detector diffractometer equipped with a graphite-monochromized Ag Kα radiation (λ = 0.56086 Å). Black crystals of the title phases were selected, cut to desired dimensions under a microscope, and mounted on MiTeGen plastic loops. To protect the crystals from air and moisture, a constant stream of cold N_2_ gas was applied during the data collection.

Data integration and absorption corrections were performed using the SAINT and SADABS software packages, respectively, as implemented in the APEX4 suite.^35,36^ The space groups were determined with the XPREP program.^37^ Crystal structures were solved using the intrinsic phasing method with SHELXT and refined by full-matrix least-squares methods on *F*^2^ with SHELXL, using the Olex2 program as the graphical interface.^38−40^

After solving the structures, the STRUCTURE TIDY program was utilized to standardize the atomic coordinates.^41^ Structures were drawn and visualized with the help of CrystalMaker software.^42^ Selected details of data collection and relevant crystallographic parameters are given in Table 1 and Supplementary Tables S1–S3. CCDC deposition numbers 2348737–2348738 contain the full supplementary crystallographic data for the compounds discussed in this paper. CIF files can be obtained free of charge by visiting https://www.ccdc.cam.ac.uk/structures/, via emailing data_request@ccdc.cam.ac.uk, or by contacting The Cambridge Crystallographic Data Centre – 12 Union Road, Cambridge CB2 1EZ, U.K., fax + 44 1223 336033.

**Table 1:** Selected Crystallographic Data and Structure Refinement Details for Eu_8_Zn_2_As_6_O and Eu_14_Zn_5_As_12_O. (*T* = 100(2) K, Ag Kα, *λ* = 0.56086 Å)

Chemical formula	Eu_8_Zn_1.88(1)_As_6_O^b^	Eu_14_Zn_5_As_12_O
fw/g mol^–1^	1804.18	3369.33
Space group	*Pbca*	*P*2/*m*
*a*/(Å)	9.0298(5)	11.2376(6)
*b*/(Å)	16.9140(8)	4.4548(2)
*c*/(Å)	22.9003(11)	16.6051(7)
*β*/°		101.1020(10)
*V* (Å^3^)	3497.6(3)	815.72(7)
*Z*	8	1
*ρ*_cal._/g cm^–3^	6.85	6.86
*μ*(Ag K_α_)/cm^–1^	222.5	223.1
Collected/independent reflections	164731/5303	24475/2740
*R*_1_ (*I* > 2σ_(*I*)_)^a^	0.0253	0.0163
*wR*_*2*_*(I > 2σ*_(*I*)_)^a^	0.0482	0.0364
*R*_1_ (all data)^a^	0.0311	0.0165
*wR*_*2*_ (all data)^a^	0.0507	0.0365
Δ*ρ*_max,min_/e^–^·Å^–3^	1.9/–1.8	1.3/–2.1
CCDC code	2348737	2348738

a *R*_1_ = Σ||*F*_o_| – |*F*_c_||/Σ|*F*_o_|. *wR*_*2*_ = {Σ[*w*(*F*_o_^2^ – *F*_c_^2^)^2^]/Σ*w*(*F*_o_^2^)^2^}^1/2^, *w* = 1/[σ^2^(*F*_o_^2^) + (*AP*)^2^ + (*BP*)], where *P* = (*F*_o_^2^ + 2*F*_c_^2^)/3; *A* and *B* are weight coefficients.

b We use the simplified Eu_8_Zn_2_As_6_O formula throughout the manuscript.

### 2.3 Elemental Microanalysis by EDX

We performed energy-dispersive X-ray spectroscopy (EDX) studies on several crystals of Eu_8_Zn_2_As_6_O and Eu_14_Zn_5_As_12_O to verify the refined compositions. We first verified the unit cells with the SCXRD method, to ensure the measurement of the correct phases. A Thermo Scientific (TFS Helios G5 PFIB CXe) scanning electron microscope equipped with an OXFORD Instruments Ultim Max Detector spectrometer was employed for the analysis of the samples. An operational acceleration voltage of 20 kV was used to collect EDX data for Eu_8_Zn_2_As_6_O and Eu_14_Zn_5_As_12_O crystals at several points and areas. Although EDX is a semiquantitative method, the obtained quantitative chemical compositions are in good agreement with the SCXRD refinements (Figure S1, Table 1). Due to the limitations of the EDX technique, we excluded oxygen from the calculations.

### 2.4 Thermoelectric Transport Property Measurements

As highlighted in Section 3.1, we attempted to fabricate phase-pure samples of Eu_8_Zn_2_As_6_O and Eu_14_Zn_5_As_12_O compounds, but our efforts were only partially successful. We could only synthesize the latter compound in bulk and with sizable single crystals suitable for transport property analysis. The temperature-dependent thermopower was measured on a needle-shaped crystal of Eu_14_Zn_5_As_12_O in both heating and cooling modes within the 300–600 K temperature range. Data were collected with a Transient Signal Technologies SB-1000 module using the integral method and a constantan wire of the same length as a reference material. The single crystal and the constantan wire were mounted on a ceramic stage using high-purity conductive silver paint (SPI Supplies). Contacts were dried in a vacuum oven at 393 K for 2 h. The stage was then placed in a temperature-variable chamber and evacuated down to 5 mTorr.

The electrical resistivity and Hall coefficient in the high-temperature (HT) interval from 300–500 K were measured under vacuum using a Transient Signal Technologies H-5000 module, utilizing the four-probe Van der Pauw technique.^43^ Four platinum wires (0.001 in.) were affixed to the crystal using the conducting silver paste. The Hall coefficient was measured with the same module using a 14-kOe field electromagnet. Low-temperature (LT) resistivity data were collected (from 240 to 300 K) using a Quantum Design Physical Property Measurement System (PPMS) with an excitation current of 10 μA on the same crystal used for the HT measurements.

### 2.5 Magnetic Property Measurements

The temperature dependence of the magnetization was measured on a DynaCool Quantum Design PPMS equipped with the Vibrating Sample Magnetometer (VSM) option. Single crystals of the Eu_14_Zn_5_As_12_O were selected under a microscope, and their unit cells were verified with SCXRD to ensure phase purity. Crystals were crushed and then enclosed in a polyethylene capsule attached to a sample holder rod. Measurements were conducted in an external field of 1 kOe across the 5–300 K temperature range in both zero-field-cooled (ZFC) and field-cooled (FC) modes. Field dependence of the magnetization was measured at 5 K, 15 K, 30 K, and 45 K over the field range from 0 Oe to 90 kOe.

### 2.6 Electronic Structure Calculations

Electronic structure calculations, conducted using the framework of the TB-LMTO-ASA code,^44^ provide deeper insights into the chemical bonding. Calculations were performed on disorder-free models with Eu_8_Zn_2_As_6_O and Eu_14_Zn_6_As_12_O compositions, as discussed in Section 3.3. The von Barth–Hedin exchange correlation functional was employed,^45^ with empty spheres introduced to satisfy the atomic sphere approximation (ASA). Eu 4f states with seven unpaired electrons were excluded or treated as core-like under the scalar-relativistic LMTO approach, considering Eu atoms as divalent species, limiting contribution to the band structure from Eu 6s and 5d orbitals. The Fermi level was selected as the energy reference (*E*_F_ = 0 eV). A basic set included Eu [6s, 5d], Zn [4s, 3d, 4p], As [4s, 4p] and O [2p] orbitals, with the Löwdin downfolding method applied to the Eu 6p, As 4d, and O 2s and 3d orbitals. Chemical bonding analysis was performed through the calculation of the energy contribution of all filled electronic states for selected atom pairs by the Crystal Orbital Hamilton Population (COHP) method, as implemented in the TB-LMTO-ASA code.^46^

## 3 Results and Discussion

### 3.1 Synthesis

Building upon the details outlined in the Experimental Section, we provide further insights pertaining to the synthesis of Eu_8_Zn_2_As_6_O and Eu_14_Zn_5_As_12_O. These compounds were initially isolated from a reaction aimed at synthesizing ternary Eu_3_ZnAs_3_ phase.^47^ The Eu–Zn–As compositional diagram is densely populated, featuring 6 reported compositions within a relatively narrow phase space, as depicted in Figure 1a.^47−52^ The projections of the Eu_8_Zn_2_As_6_O and Eu_14_Zn_5_As_12_O compositions align closely with that of the Eu_3_ZnAs_3_ compound, illustrating a near compositional match (Figure 1a). It is not surprising that the minuscule amounts of O in the Eu/As rich reaction mixture appear to facilitate the formation of these quaternary pnictide oxide phases, which are compositionally closely related to the corresponding ternary phases (Figure 1b).

As mentioned in the Experimental Section, despite the absence of oxygen (in an elemental form or in a compound) among the reactants, the reproducibility of the results was surprisingly high, even when starting materials were sourced from different suppliers. However, the exact source of oxygen remains unidentified. In prior works, the serendipitous formation of pnictide oxides has been observed, for example in the cases of *AE*_5_*M*_2_As_5_O (*AE* = Ba, Eu; *M* = Zn, Cd; *Pn* = As, Sb),^1,34,53,54^ U_2_Cu_2_As_3_O,^55^ and Ba_5_Cd_2_Sb_4_O_2_,^56^ to name a few. Our initial hypothesis was based on the reported studies, where alumina crucibles are the oxygen source via a reduction process with the rare-earth metal.^57^ However, experiments conducted in Nb and BN crucibles under identical conditions also yielded title oxypnictides, dismissing the possibility that alumina crucibles act as the oxygen source. This indicates that oxygen is likely coming into the reaction mixture through inadvertent partial oxidation of one of the metallic elements, for instance, during the ampule-sealing process.

Attempts to make phase-pure Eu_8_Zn_2_As_6_O and Eu_14_Zn_5_As_12_O materials using various oxides, such as Eu_2_O_3_, As_2_O_3_, and ZnO, or by substituting the flux metal to Sn and Bi, predominantly resulted in known ternary and quaternary arsenides, such as Eu_14_Zn_1+x_As_11_,^48^ Eu_21_Zn_4_As_18_,^49^ and Eu_11_Zn_4_Sn_2_As_12_,^58,59^ suggesting the staring materials or the flux metal may introduce reactive oxygen. Given that the high declared purity of reactants is typically metal-based, we cannot exclude the presence of oxide/hydroxide impurities in catalyzing oxypnictide formation. For instance, we identified the presence of a small amount of As_2_O_3_ impurity in the used arsenic from the PXRD experiment.

Synthetic conditions described in Section 2.1 with reduced cooling rates yielded sizable single crystals of Eu_14_Zn_5_As_12_O (up to 1.5 mm) suitable for property measurements. Efforts to grow larger single crystals of Eu_8_Zn_2_As_6_O were unsuccessful, limiting our study of this novel material to structural and computational descriptions, although its unique complex crystal structure motivates us to explore its magnetic and transport properties in the future. Both compounds exhibit moderate stability in ambient conditions and do not deteriorate, should they be exposed to air for several days.

The morphology and the appearance of the crystals of both compounds are hardly distinguishable, featuring black block-shaped and needle-shaped crystals, although the observed needles of Eu_14_Zn_5_As_12_O are typically longer. Generally, we almost always obtain both compounds simultaneously, though Eu_14_Zn_5_As_12_O tends to predominate quantitatively. This prevalence notably complicates the crystal selection process for property studies.

### 3.2 Crystal Chemistry of Eu_8_Zn_2_As_6_O and Eu_14_Zn_5_As_12_O

The newly discovered quaternary arsenide oxide phases adopt novel structure types—a consequence attributed to the unique integration of Zn cations and multiple anions with varying coordination geometries influenced by their size, charge, and electronic configurations. A literature survey indicates that the crystal structures of multinary oxypnictides often establish their own structure types, offering a robust playground for exploring structure–property relationships within this class of materials.^1^

Both compounds are highly disordered, primarily within the framework of Zn atoms, which are known to display different coordination preferences, such as tetrahedral, square-planar, and trigonal-planar, within the realm of Zintl pnictides and oxypnictides.^47,48,59−63^ Another similarity between these compounds is the coordination environment of the O^2–^ anions, which are nested among six Eu^2+^ cations, composing a chain of corner-sharing [Eu_6_] octahedra oriented along the shortest lattice vector in both structures. Despite their compositional proximity (Figure 1b), the structures of Eu_8_Zn_2_As_6_O and Eu_14_Zn_5_As_12_O are distinctly different. In the following paragraphs, we will provide a brief discussion of each crystal structure, underscoring the role of disorder in achieving charge-balanced compositions.

#### 3.2.1 Crystal Structure of Eu_8_Zn_2_As_6_O

**Figure 2:**
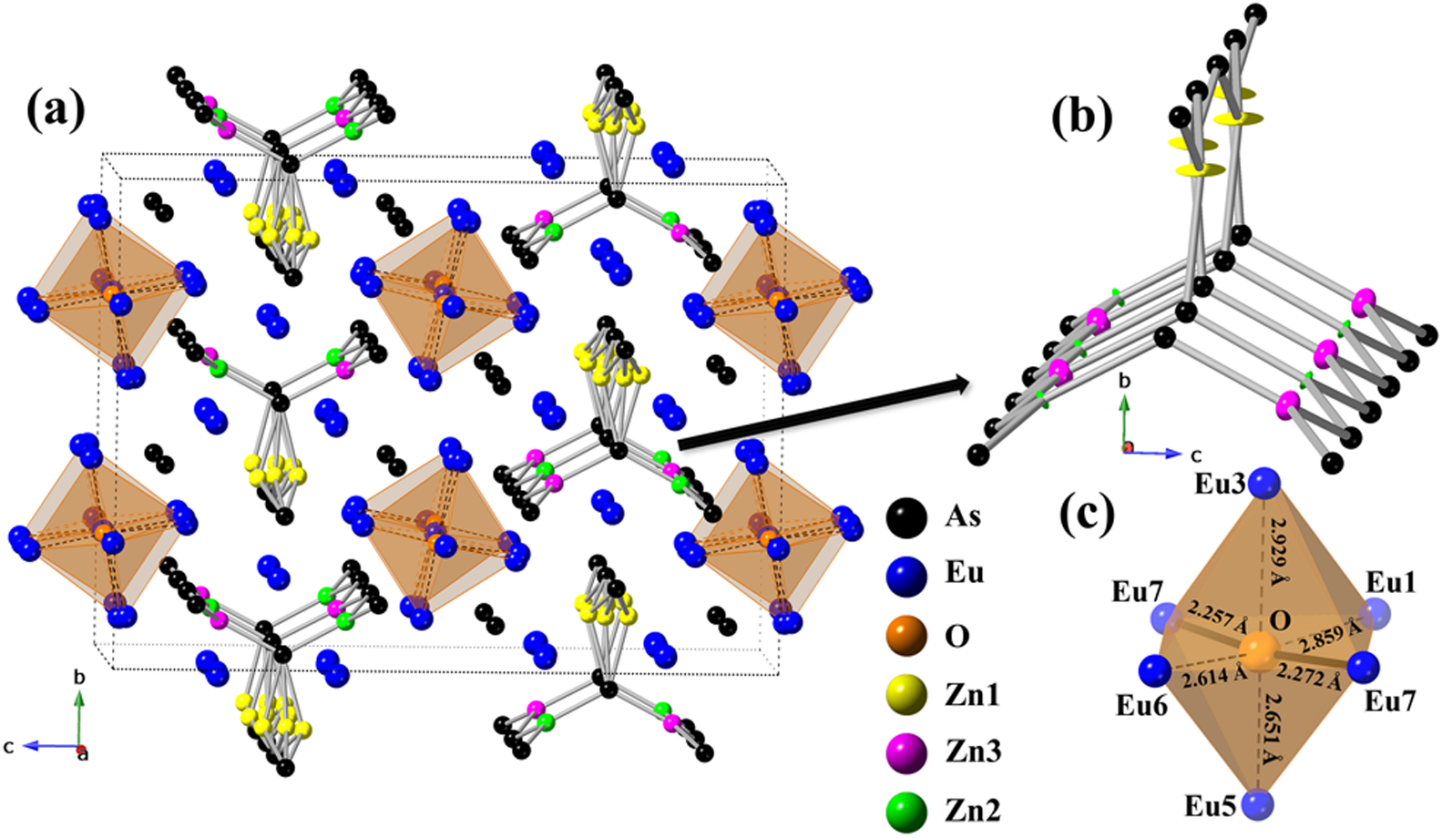
(a) Crystal structure of Eu_8_Zn_2_As_6_O, viewed along the crystallographic *a*-axis. Unit cell is outlined with dotted lines. As, Eu, O, Zn1, Zn2, and Zn3 atoms are displayed as black, blue, orange, yellow, green, and pink spheres, respectively. (b) Anionic substructure with the Zn1 atom not modeled positionally. Ellipsoids are drawn with a 50% probability. (c) The local coordination environment of the oxygen atom.

Eu_8_Zn_2_As_6_O crystallizes in the orthorhombic space group *Pbca* (No. 61) with 8 formula units per unit cell. Its crystal structure is heavily disordered, with multiple split Eu and Zn atomic positions (Figure 2a, Table S1). However, a simplified ordered model offers a better initial understanding of the unique structure and the chemical bonding, thus we will first describe a disorder-free model, gradually introducing specific details of the structural complexity.

During the initial quality screening of diffraction data, the structure was tentatively solved in the space group *Cmcm* with unit call parameters *a* = 4.52 Å, *b* = 16.93 Å, and *c* = 22.90 Å. However, the synthesized precession images indicated that this indexing of the diffraction pattern did not account for all observed reflections. A subsequent analysis using a more powerful X-ray source identified a superstructure with a doubled lattice parameter *a* (Table 1). This solution yielded an asymmetric unit with eight crystallographically independent Eu sites, three Zn sites, six As sites, and one O site, all occupying 8*c* general positions, as specified in Table S1. The atomic positions were assigned based on peak height, coordination environment, and bond length analysis, although subsequent structure refinement revealed significant deficiencies in this initial model.

The disorder-free representation of this structure showcases all 18 atomic sites as fully occupied (Figure 2b). This model yields a composition Eu_8_Zn_3_As_6_O, which shows an excess of two electrons, i.e., (Eu^2+^)_8_(Zn^2+^)_3_(As^3–^)_6_(O^2–^)(*e*^–^)_2_. Close examination of the Fourier difference map revealed the presence of noticeable holes at Zn2 and Zn3 sites, which were subsequently modeled as partially occupied, with site occupancy factors (SOFs) of ca. 0.75 and 0.13, respectively (Table S1). The refinement yielded a nearly stoichiometric Eu_8_Zn_1.88(1)_As_6_O composition, in excellent agreement with the charge-balance considerations and EDX results (Figure S1). The underoccupancy of these Zn sites is apparently not rooted in geometric considerations since the interatomic Zn—As distances and the Zn trigonal-planar coordination are reasonable.^47,48,63,64^ Therefore, the underoccupancy of the Zn sites must be explained by the drive to maintain the charge-balanced composition. This is similar to the case of Ca_9_Zn_4.5_Sb_9_.^65^

In the simplest terms, the new structure can be broken down to eight Eu^2+^ cations, one O^2–^ anion, and a [Zn_2_As_6_]^14–^ polyanion comprised of tri-star shaped infinite ribbons of corner-sharing trigonal planar [ZnAs_3_] units (Figure 2b). These units, interconnected through As2 atoms and running along the [100 direction], present the same structural motif reported in the Ca_9_(Zn_1-x_In_x_)_4_Sb_9_ phase, although the tri-star polyanionic fragment in the latter is composed of symmetry equivalent [ZnSb_3_] units.^64^ A notable structural feature of the [Zn_2_As_6_]^14–^ anions is the alteration of corner-sharing [Zn2As_3_] and [Zn3As_3_] units centered by partially occupied Zn sites within two out of the three ribbons (Figure 2b).

Further analysis of the crystal structure highlighted a noticeably elongated atomic displacement parameter (ADP) for the Zn1 site oriented perpendicularly to the [Zn1As_3_] plane. Additionally, two significant residual electron density peaks were located within 0.6–0.8 Å, suggesting positional disorder at this site. Addressing these peaks as partially occupied Zn atoms led to the identification of three split positions (labeled as Zn1A, Zn1B, and Zn1C), summing to a total occupancy of 1, which was subsequently constrained in the presented structural model (Table S1). Notably, these split Zn sites slightly protrude from the [As_3_] plane, akin to the coordination of interstitial Zn atoms observed in the Ca_9_Zn_4+*x*_Sb_9_ phase, offering insight into the structural versatility of Zn within this framework.^65^ The degree of protrusion can be estimated from the sum of the three As–Zn–As angles, which are ca. 343.7°, 358.5°, and 336.7° for Zn1A, Zn1B, and Zn1C, respectively. Notably, the corresponding sums of the bond angles for the nearly ideal trigonally planar coordinated Zn2 and Zn3 atoms are 359.1° and 358.9°, respectively, being close to the ideal 360° value.

The observed Zn–As interatomic distances range approximately from 2.44 Å to 2.70 Å, with the longest distances linked to the protruding Zn atom, are comparable to those in related Zintl arsenides and oxyarsenides, such as Eu_3_ZnAs_3_ (2.47–2.71 Å),^47^ Eu_11_Zn_4_Sn_2_As_12_ (2.49–2.50 Å),^59^ Ba_2_Zn_3_As_2_O_2_ (2.58 Å),^60^ Eu_5_Zn_2_As_5_O (2.56–2.69 Å),^34^ K_2_Zn_5_As_4_ (2.45–2.66 Å),^66^ and Eu_2_Zn_2_As_3_ (2.54–2.65 Å),^50^ reflecting the range of Zn coordination geometries.

The O^2–^ anions are located within the octahedral voids formed by six Eu^2+^ cations (Eu1, Eu3, Eu5, Eu6, and Eu7 sites), composing an O-centered distorted corner-sharing [Eu_6_] octahedra and thus forming 1D chains nested around the discussed tri-star polyanionic zinc-arsenide ribbons in a checkerboard pattern (Figure 2a). A detailed bonding analysis uncovers significant variations in interatomic distances within the [OEu_6_] polyhedron (Figure 2c, Table S3). The Eu–O contacts in the plane perpendicular to the [100] direction are notably longer (2.65–2.95 Å) compared to those aligned along this direction (2.26–2.27 Å) (Figure 2c). Considering the sum of the ionic radii for Eu_(CN = 6)_^2+^–O_(CN = 2)_^2–^ and Eu_(CN = 6)_^3+^–O_(CN = 2)_^2–^ of ca. 2.52 Å and 2.30 Å, respectively, it is more accurate to view the europium oxide sublattice as a linear chain of Eu7–O bonds.^67^ This interpretation is supported by the observed coordination environments of the Eu sites (Figure S2). The Eu atoms not bonded to O (Eu2 and Eu4, and Eu8) form slightly distorted [EuAs_6_] octahedra, whereas Eu1, Eu3, Eu5, and Eu6 atoms are coordinated by five As atoms in a square pyramidal fashion. These polyhedra can be extended to the distorted octahedra by including O atoms, though these extended Eu–O contacts are too lengthy to be viewed as bonds, *vide supra*. In contrast, the Eu7 site is encircled by two O atoms and four As atoms, forming an almost perfect octahedron with O–Eu–As and As–Eu–As angles close to 90° (Figure S2g).

Positional disorder was observed for most of the Eu cationic sites. To achieve a nearly featureless Fourier difference map, we modeled observed residual electron density peaks located near Eu1, Eu2, Eu3, and Eu4 atoms as split sites, while maintaining the total Eu content (Table S1). Such extensive disorder among Eu and Zn sites likely has a significant impact on transport properties by enhancing phonon scattering. We speculate that this contributes to relatively low thermal conductivity, akin to observations for Yb_10_MnSb_9_ and Yb_21_Mn_4_Sb_18_ phases.^24,68,69^

#### 3.2.2 Crystal Structure of Eu_14_Zn_5_As_12_O

Eu_14_Zn_5_As_12_O crystallizes in the centrosymmetric space group *P*2/*m* (No. 10) of the monoclinic crystal system with one formula unit per unit cell. This novel quaternary oxyarsenide shares some structural similarities with Eu_8_Zn_2_As_6_O, yet their anionic substructures differ significantly. The initial structure solution yielded an asymmetric unit with eight crystallographically independent Eu sites, three Zn sites, seven As sites, and one O site, all occupying special positions (Table S2). Similar to the Eu_8_Zn_2_As_6_O scenario, the initially refined (Eu^2+^)_14_(Zn^2+^)_6_(As^3–^)_12_(O^2–^)(*e*^–^)_2_ composition is unbalanced, presuming all identified atomic positions are fully occupied. This structural model can be seen in Figure S5. However, partial occupancy for Zn2 (Wyckoff 2*m*) and Zn3 (Wyckoff 2*n*) sites became evident, with the freed refined site occupancy factors (SOFs) of. ca. 0.75. Constraining their occupancies to this figure allowed us to attain a charge-balance composition of Eu_14_Zn_5_As_12_O (Table 1).

The notably elongated ADPs revealed positional disorder in all Zn sites. Unlike the Zn1 site in the Eu_8_Zn_2_As_6_O phase, which indicates a similar issue, Zn sites in Eu_14_Zn_5_As_12_O split into two partially occupied positions. Their freed combined occupancies sum to ca. 1 for Zn1A+Zn1B and ca. 0.75 for Zn2A+Zn2B and Zn3A+Zn3B, leading us to constrain their occupancies accordingly to maintain the chare-balanced composition.

**Figure 3:**
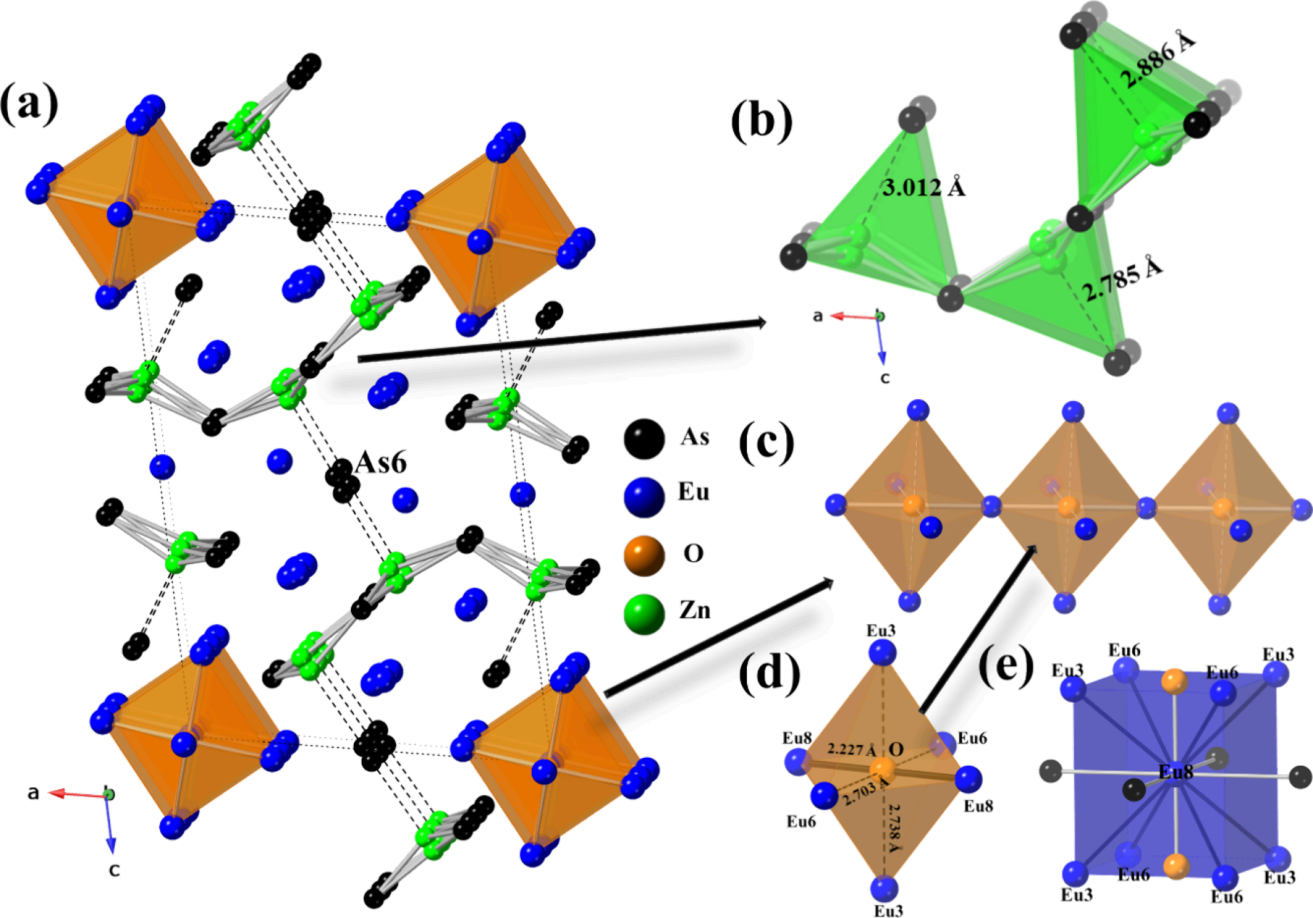
(a) Crystal structure of Eu_14_Zn_5_As_12_O, viewed along the crystallographic *b*-axis. The unit cell of Eu_14_Zn_5_As_12_O is outlined with dotted lines. As, Eu, O, and Zn atoms are displayed as black, blue, orange, and green spheres, respectively. (b) Polyanionic substructure consisting of trigonal-planar [ZnAs3] units. Note significantly elongated Zn–As contacts completing tetrahedral coordination. (c) A linear chain of octahedral O-centered corner-sharing [Eu_6_]. (d) The local coordination environment of an oxygen atom in Eu_14_Zn_5_As_12_O with highlighted bond distances. (e) [Eu8As_4_O_2_] octahedral and [Eu8Eu_8_] cubic coordination of a Eu8 atom. Noticeably short Eu8–Eu3/Eu6 bond distances are observed within the [Eu_8_] cube.

All split Zn sites fall into two distinct groups. First, Zn1B, Zn2B, and Zn3B atoms, holding roughly one-third of the total occupancy for their corresponding Zn*X*A+Zn*X*B pairs, are coordinated by three As atoms in a nearly ideal trigonal planar fashion (with the sum of As–Zn*X*B–As around 357–360°). In contrast, Zn1A, Zn2A, and Zn3A atoms slightly protrude from the [As_3_] plane, as indicated by the sum of As–Zn*X*A–As angles ranging from ca. 338–348°. This minor deviation from planarity is insufficient to classify the coordination environment as distorted tetrahedral, given the very long axial Zn–As contacts. Nonetheless, large thermal parameters for As6 (Wyckoff 1*h*) and As7 (Wyckoff 1*d*) atoms, aligned toward the split Zn2 and Zn3 atoms, respectively, indicated further As6 and As7 disorder. Upon moving them to 2*m* and 2*n* Wyckoff sites, a smoother and more uniform Fourier difference map was obtained. This doubling of the site multiplicity necessitated halving the occupancy for the arsenic atoms, mirroring the approach used to model the [*Pn*_3_]^7–^ (*Pn* = P, As) linear unit in numerous 14–1–11 phases.^27,48,70^ The refined interatomic distances between Zn2A–As6 and Zn3A–As7 are notably reduced (ca. 2.79 Å and 2.89 Å, respectively) compared to those in the initial structural model, yet they remain somewhat longer than the sum of the covalent radii of Zn and As.^71^ The overall arrangement of Zn-centered polyhedra can be viewed as 1D “ribbonlike” chains made up of three corner-sharing [ZnAs_4_] tetrahedra and [ZnAs_3_] trigonal planar units oriented along the [010] direction (Figure 3a).

The dimensionality of these chains could potentially be expanded through iso- and aliovalent doping of Zn atoms with transition or main group metals that prefer tetrahedral coordination, such as Mn or Al. This type of doping might increase the separation between ZnA and ZnB sites, promoting tetrahedral coordination and linking the ribbons along the *c*-axis. This strategy was successfully applied to the *AE*_3_Zn*Pn*_3_ (*AE* = Sr, Eu; *Pn* = P, As) family of compounds, aiding in the modulation of transport properties by enhancing both electrical conductivity and the Seebeck coefficient.^47^

The coordination environment of oxygen anions resembles the case of Eu_8_Zn_2_As_6_O, *vide supra*, with O^2–^ located within an octahedral hole of six Eu^2+^ cations (Eu3, Eu6, and Eu8 sites). Only Eu8–O contact of ca. 2.28 Å falls within the bonding range, whereas the Eu–O and Eu6–O interatomic distances extend beyond 2.70 Å (Figure 3d). A closer examination of the local coordination environment of the Eu atoms indicates a distorted octahedral or square pyramidal coordination of As atoms, except for Eu8, which is octahedrally coordinated by four As atoms and two O atoms (Figure S3). In line with the structure of Eu_8_Zn_2_As_6_O, the europium oxide sublattice is best described as a linear chain of Eu8–O bonds (Figure 3c). Additionally, two Eu sites exhibit positional disorder and were modeled similarly to the Eu atoms in the Eu_8_Zn_2_As_6_O structure, *vide supra*, thereby maintaining a balanced composition.

Ultimately, we would like to discuss the impact of structural disorder and bonding on charge balance. The underoccupancy of Zn2 and Zn3 sites in both compounds is a prerequisite for attaining charge-balanced compositions, denoted as (Eu^2+^)_8_(Zn^2+^)_2_(As^3–^)_6_(O^2–^) and (Eu^2+^)_14_(Zn^2+^)_5_(As^3–^)_12_(O^2–^), based on a fully ionic approximation. This underpins the necessity for the Eu atoms to adopt a Eu^2+^ state, leading to the semiconducting behavior—a prediction supported by our calculations and experimentally demonstrated for Eu_14_Zn_5_As_12_O *via* electrical resistivity measurements (see Figure 6a below). However, the notably shorter Eu–O distances of 2.26–2.28 Å are typically reported for compounds containing trivalent Eu^3+^ species.^72^ One might theorize the occurrence of mixed-valence states for Eu atoms as a plausible explanation for the refined Zn-deficient composition of Eu_8_Zn_1.88(1)_As_6_O, yet the limited amount of material available precluded the measurement of magnetic properties for this compound. Furthermore, the bonding analysis for [EuO_2_As_4_] octahedra does not reveal the significant shortening of Eu8–As contacts, arguing against the presence of mixed-valence Eu^2+^/Eu^3+^ states.

Finally, the magnetic properties of Eu_14_Zn_5_As_12_O are discussed in section 3.5. Although a slightly reduced magnetic moment was observed, inconsistent with expectations for Eu^2+^, this discrepancy is attributed to experimental uncertainty.

### 3.3 Electronic Structure and Chemical Bonding

The majority of rare-earth-bearing Zintl pnictides and oxypnictides are typically formed by divalent lanthanide species, such as Eu^2+^ and Yb^2+^.^1,21^ To corroborate the anticipated charge-balanced configurations and delve deeper into the chemical bonding of these materials, electronic structure calculations were conducted for both compounds. Spin–orbit coupling and the presence of highly localized 4f electrons cause significant challenges for the accurate analysis of lanthanide-containing compounds. To negate some of these effects, 4f orbitals were either excluded from the calculations or treated as core states in Eu_8_Zn_2_As_6_O and Eu_14_Zn_5_As_12_O, thus minimizing the influence of half-filled 4f orbitals, as expected for Eu^2+^ species. This approach is widely used for Eu- and Yb-containing compounds, providing a reliable basis for preliminary analysis of the electronic structure.^34,73−77^

**Figure 4:**
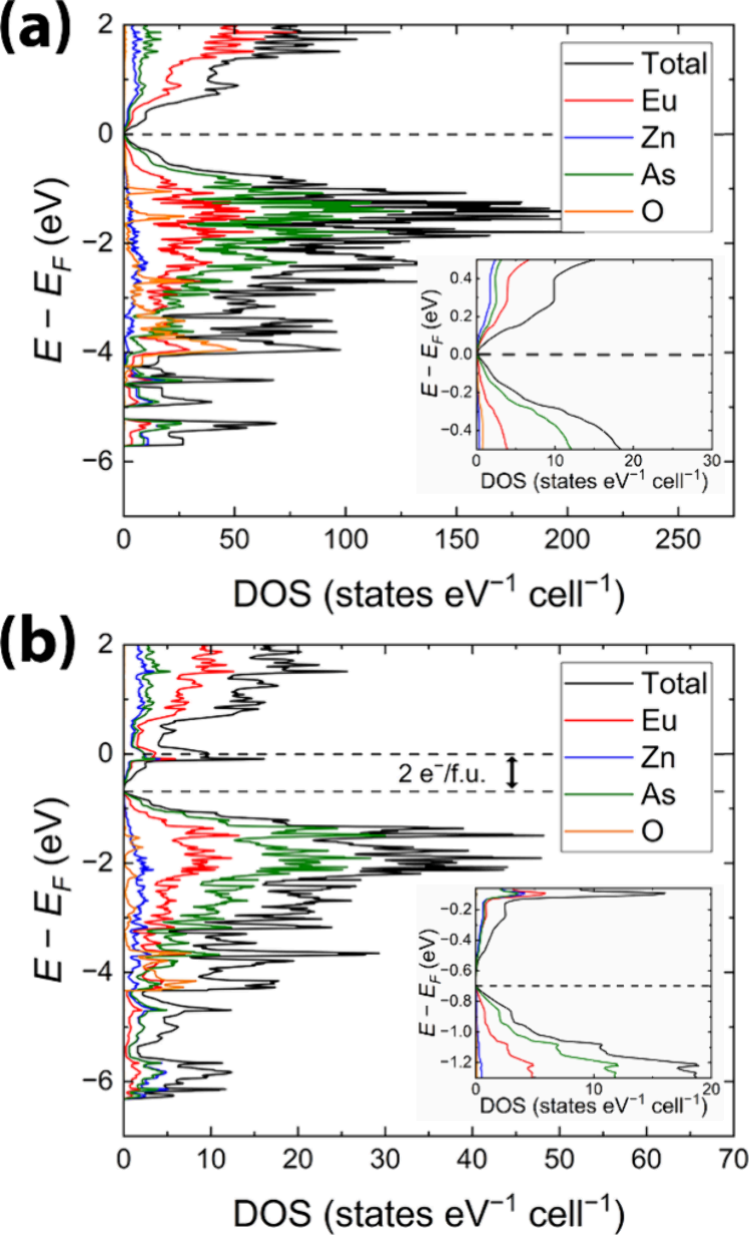
Calculated Total Density of States (TDOS) plots together with partial contributions for all atoms (PDOS) for (a) Eu_8_Zn_2_As_6_O and (b) idealized Eu_14_Zn_6_As_12_O structures. The Fermi level is the energy reference at 0 eV. Insert panels exhibit the close-up view of the band gap, VBM, and CBM.

**Figure 5:**
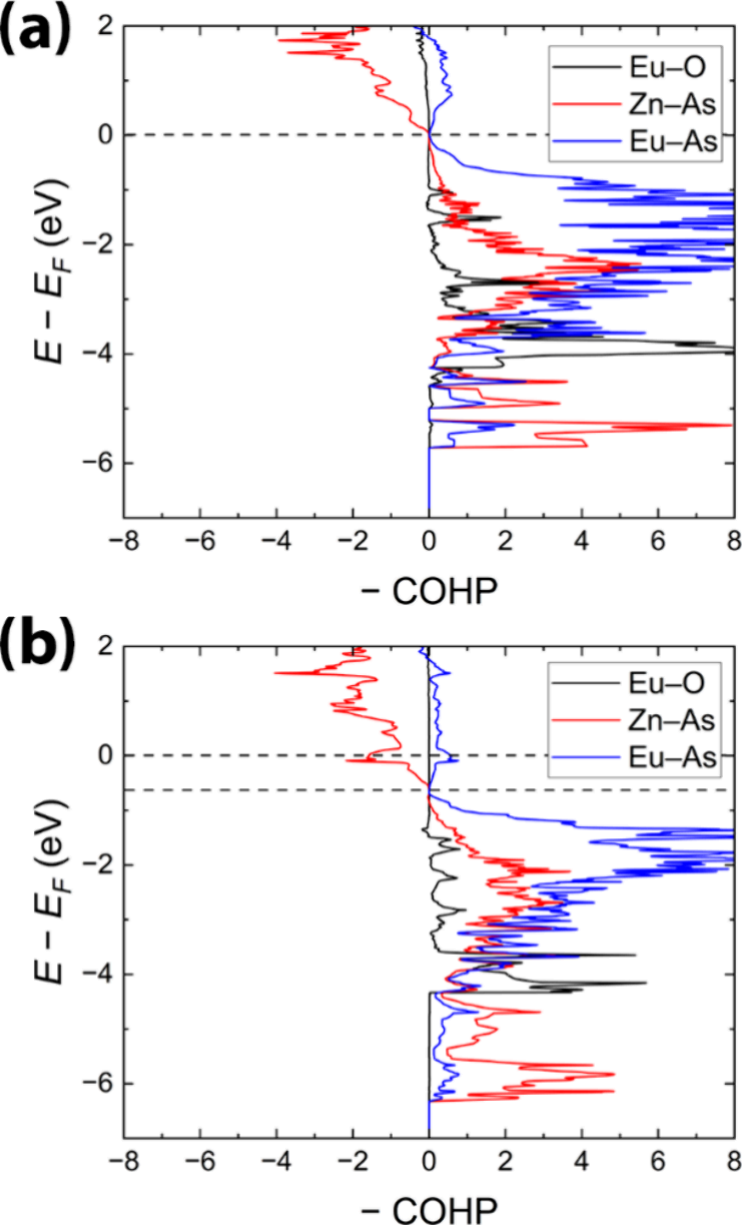
Calculated cumulative COHP curves for cumulative Eu–O, Zn–As, and Eu–As interatomic contacts in (a) Eu_8_Zn_2_As_6_O and (b) Eu_14_Zn_6_As_12_O. The Fermi level is the energy reference at 0 eV. An additional dashed line at ca. −0.69 eV indicates a 2-election shift corresponding to the Eu_14_Zn_5_As_12_O composition.

For Eu_8_Zn_2_As_6_O, the calculations utilized a simplified structural model made by adjusting the occupancy of the Zn3 site to 0 and preserving the full occupancy of Zn2. This disorder-free electron-precise model accurately represents the anionic substructure and the stoichiometric Eu_8_Zn_2_As_6_O composition (Figure S4) and, though simplified, perfectly fits for the purposes of calculating electronic structure. The electronic density of states (DOS) curves for Eu_8_Zn_2_As_6_O are shown in Figures 4a and S6. We observed the presence of a tiny band gap of 0.02 eV, which supports the charge-balanced notation, *vide supra*. The DOS analysis indicates that As-p orbitals predominantly contribute to the states below the valence band maxima (VBM), followed by the contribution from Eu-d and Eu-p orbitals, with negligible contribution from Zn and O orbitals. As can be seen in Figure S6, the Eu atoms exhibit a distinct separation of s, p, and d orbitals, with Eu-s orbitals dominating the region from –5 eV to –3 eV, while Eu-p orbitals largely contribute to the area from –2 eV to –1 eV. Interestingly, O-p orbitals contribute significantly to the same energy range as the Eu-s orbitals, highlighting the crucial role of their interaction in forming Eu–O bonds, as further evidenced by the COHP analysis (Figure 5a). We also observe a clear distinction between Zn-s (−5.5 eV to – 4.5 eV) and Zn-p (−4 eV to VBM) orbitals, which indicates their poor hybridization.

The TDOS and PDOS plots for Eu_14_Zn_6_As_12_O (Figure 4b, S6) exhibit nearly identical patterns to those of Eu_8_Zn_2_As_6_O. However, the Fermi level appears to fall in an area with relatively high DOS and strong antibonding character of Zn–As interactions, as can be seen from the COHP curves (Figure S7b). This observation can be explained by the disorder-free structural model used for the calculations (Figure S5). Since the SOFs for two Zn sites in Eu_14_Zn_5_As_12_O were refined to 0.75 (Table S2), constraining their occupancies to 1 results in a 2-electron-rich model with a composition of Eu_14_Zn_6_As_12_O(*e*^–^)_2_, as discussed in Section 3.2.2. Indeed, a shift of two electrons per formula unit from the Fermi level at – 0.69 eV is noted in Figure 4b, revealing a small band gap of 0.02 eV. Similar behavior is observed in Eu_5_*M*_2_As_5_O (*M* = Zn, Cd) and in numerous *A*_14_*MPn*_11_ and *A*_10_*M*_6_Pn_12_ phases (*A* = Ca, Sr; Eu, Yb; *M* = Mg, Cd, Zn; *Pn* = As, Sb, Bi).^34,48,77−80^ Band gaps in both Eu_8_Zn_2_As_6_O and Eu_14_Zn_6_As_12_O are likely underestimated, due to the limitations of the LMTO code and because the ordered models were not geometrically optimized. Our transport property measurements align with these findings, *vide infra*.

By examining the COHP plots (Figure 5), we can infer important information about the chemical bonding of the structures. With two electrons per formula unit removed from the disorder-free Eu_14_Zn_6_As_12_O composition, the Fermi level shifts toward the valence band and is now found in a gap, providing a more reasonable basis for bonding analysis. As demonstrated in Figure 5b, the average Zn–As, Eu–As, and Eu–O contacts in both compounds are optimized and show bonding interactions below the Fermi level. The Zn–As contacts in both compounds are characterized by a certain degree of covalency, with the negative integrated COHP (−ICOHP) values for symmetrically independent pairs ranging from 1.21 eV/bond to 1.81 eV/bond. The −ICOHP values for comparable bonds within trigonal planes in Eu_8_Zn_2_As_6_O are slightly larger, 2.18 eV/bond and 2.24 eV/bond, pointing to the greater covalent interaction within the polyanionic substructure. Conversely, the reduced values in Eu_14_Zn_6_As_12_O may stem from an unoptimized basis and, consequently, slightly enlarged Zn–As contacts. The longest Zn–As contacts, complementing the polyhedra to tetrahedral geometry, exceed 3.0 Å (Zn1–As1, Zn2–As6, and Zn3–As7) in the Eu_14_Zn_6_As_12_O model (Figure S5), exhibiting minimal bonding activity compared to shorter Zn–As bonds (averaged −ICOHP is 0.3 eV/bond). This suggests a preference for trigonal planar coordination of Zn, with significantly lower calculated −ICOHP values for these extended contacts, between 0.17 eV/bond and 0.50 eV/bond, indicating negligible interaction.

Short Eu–O contacts (2.27 Å in Eu_8_Zn_2_As_6_O and 2.23 Å in Eu_14_Zn_6_As_12_O) are optimized, signifying strong interactions with calculated −ICOHP values of 1.14 eV/bond for Eu7–O in Eu_8_Zn_2_As_6_O and 1.29 eV/bond for Eu8–O in Eu_14_Zn_6_As_12_O (Figure S7). The areas corresponding to bonding regions align closely with contributions from O-s and Eu-s/Eu-d orbitals, highlighting significant covalency for these interactions (Figure S6). Longer Eu–O contacts demonstrate lower −ICOHP values around 0.5 eV/bond (Figure S7), which points to much weaker but still appreciable orbital mixing/interactions. This supports the notion that oxygen is situated in an octahedral void among six Eu atoms, yet with limited Eu–O bonding interaction.

### 3.4 Transport Properties of Eu_14_Zn_5_As_12_O

Both title Eu-bearing oxyarsenides are thermally stable and chemically inert compared to typically air-sensitive alkali or alkaline-earth metal-based Zintl phases, which makes them suitable candidates for property studies and further exploration of structure–property relationships. The closest structurally related compounds to Zintl oxyarsenides are their oxygen-free counterparts – Zintl arsenides. Although semiconducting properties of Zintl oxyarsenides are not well established, being limited to a few structure types,^1,81,82^ compositionally simpler Eu-based Zintl arsenides are known for their exotic physical properties, such as colossal magnetoresistance, complex magnetic ordering, and thermoelectric performance.^59,75,83−88^ Given their similar structural chemistry, heteroanionic Zintl oxypnictides also present a promising avenue for probing fascinating physical phenomena, thereby justifying the research efforts. As discussed in Section 3.1, we obtained sufficiently large single crystals of the Eu_14_Zn_5_As_12_O, which were suitable for physical property studies. In the following paragraphs, we will provide a comprehensive discussion of the thermoelectric and magnetic properties of this novel compound.

#### 3.4.1 Thermopower

**Figure 6:**
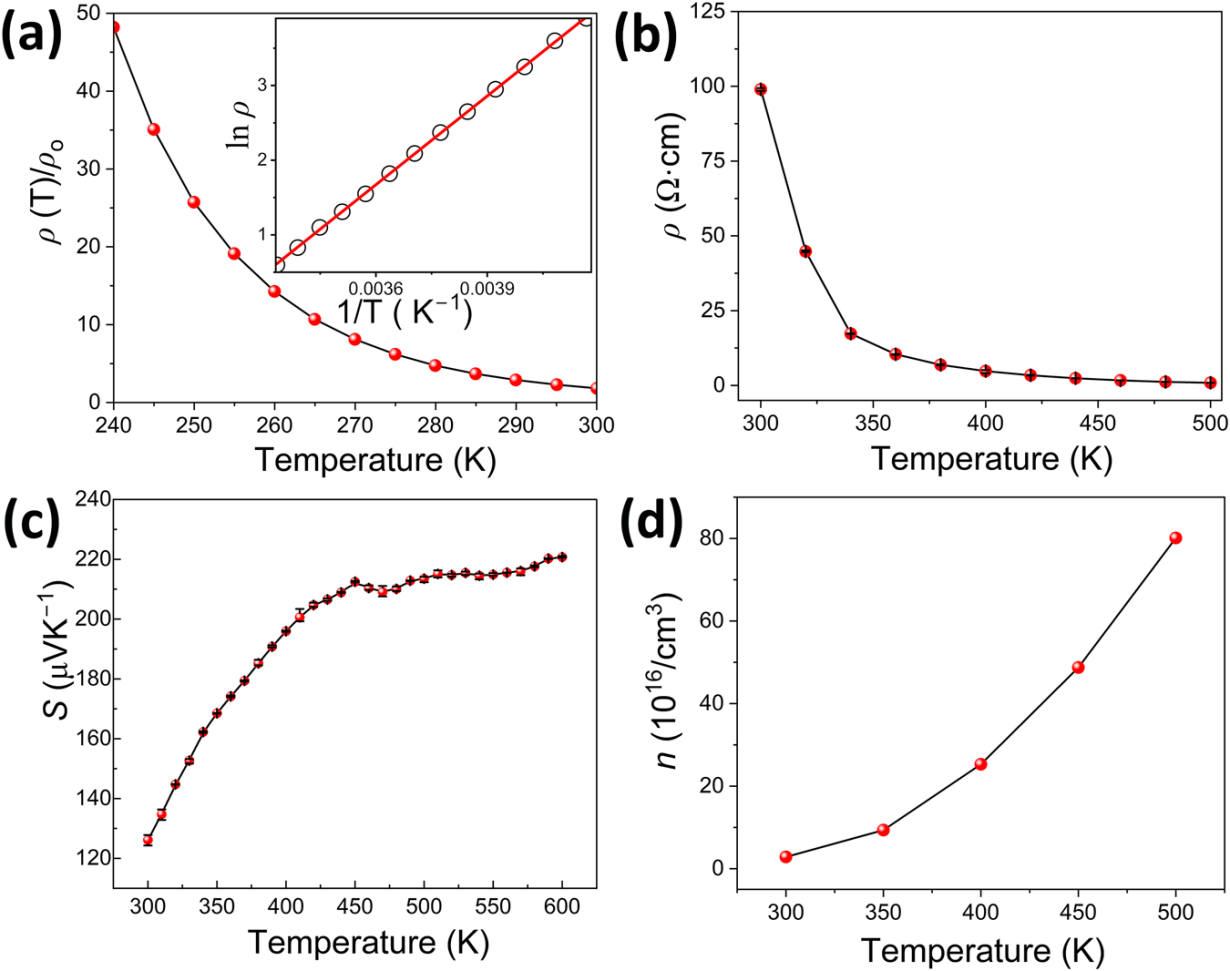
(a) Temperature-dependent resistivity measured on a single crystal of Eu_14_Zn_5_As_12_O in the low-temperature mode. The inset shows ln *ρ* vs 1/*T* plot, with the red solid line representing a linear fit to the Arrhenius equation. (b) Temperature dependence of electrical resistivity in the high-temperature region. (c) Temperature dependence of the Seebeck coefficient *S* for a single crystal of Eu_14_Zn_5_As_12_O. (d) Variation of charge carrier concentration on heating the sample from 300 to 500 K, as determined from Hall effect measurements.

The temperature-dependent variation of thermopower, *S*, was measured on a needle-like single crystal of Eu_14_Zn_5_As_12_O across the temperature range of 300 to 600 K, as depicted in Figure 6c. The Seebeck coefficient values are large and positive throughout the measured interval, hinting at the holes being the majority charge carriers in Eu_14_Zn_5_As_12_O and indicating p-type semiconducting behavior. This observation corroborates well with the Hall coefficient measurements, as discussed below. The Seebeck coefficient values increase parabolically from 300 to 450 K with the values of 126 μV K^–1^ and 212 μV K^–1^, then plateauing up to 600 K with a marginal increase, achieving a maximum of 220 μV K^–1^ in the measured range. A cooling cycle confirmed the consistency of thermopower values, supporting the accuracy of the data.

The Seebeck coefficient (eq 1) for metallic and degenerate semiconducting materials is proportional to the temperature (*T*) and effective mass (m*) and inversely proportional to the charge-carrier concentration (*n*) and is described by the relatively simple model:
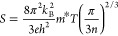
1where *k*_B_, *h*, and *e* represent Boltzmann’s constant, Planck’s constant, and electronic charge, respectively.^25^ It is expected that materials with low carrier concentrations, such as insulators and semiconductors, should exhibit large Seebeck coefficients. A high thermopower (*S*) also implies a large effective mass, corresponding to a large density of states (DOS), consistent with our electronic structure calculations (Figure 4b).^89^ Conversely, low electrical resistivity requires a large charge-carrier concentration and mobility (*μ*), which can be expressed as:
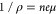
2

Since charge carrier concentration affects both Seebeck and electrical resistivity, the thermopower and resistivity values are typically related.^90^ As temperature rises, a simultaneous increase in electrical conductivity, thermopower, charge-carrier concentration, and mobility is observed—characteristic of intrinsic semiconductors (Figure 6).

Although thermoelectric studies on structures similar or identical to Eu_14_Zn_5_As_12_O are scarce, the measured Seebeck coefficients for Eu_14_Zn_5_As_12_O compare favorably with those observed in other semiconducting Zintl pnictides and oxypnictides. The high *S* values were observed in the *RE*_2_SbO_2_ (*RE* = Nd, Sm, Gd, Ho, and Er) oxyantimonides (∼210 μV K^–1^ at 400 K),^29^ Sr_3_ZnAs_3_ (∼250 μV K^–1^ at 550 K),^47^ Sr_3_AlSb_3_ (∼470 μV K^–1^ at 800 K),^91^ Ca_5_Al_0.95_In_0.95_Zn_0.1_Sb_6_ (∼200 μV K^–1^ at 900 K),^92^ and the structurally akin Ca_9_Zn_4+x_Sb_9_ phase (∼270 μV K^–1^ at 873 K).^28^

#### 3.4.2 Electrical Transport Properties

Electrical resistivity in LT (low-temperature) and HT (high temperature) modes was measured on the same single crystal of the Eu_14_Zn_5_As_12_O phase used for thermopower measurements. The variation of electrical resistivity *ρ*(*T*) with temperature is depicted in Figure 6. The *ρ* values decrease upon heating, indicative of the semiconducting nature of the disordered Eu_14_Zn_5_As_12_O phase. Resistivity drops from ca. 48 Ω·cm at room temperature to 8 Ω·cm at 500 K, significantly exceeding the magnitude expected for decent thermoelectric materials. Such elevated resistivity is expected for structurally and compositionally related undoped Zintl pnictides, such as Sr_3_ZnAs_3_, Eu_5_Al_2_Sb_6_, Sr_3_AlSb_3_, Sr_5_In_2_Sb_6_, etc.^47,91,93,94^ LT resistivity data gathered using a PPMS is consistent with the HT data, demonstrating an exponential increase upon cooling down to 240 K (Figure 6a). An intrinsic band gap of 0.68 eV was estimated by fitting the data to equation 3, where *E*_g_, *k*_B_, and *T* stand for the band gap, Boltzmann constant, and temperature, respectively.
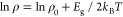
3

The fitted value confirms the semiconducting nature of Eu_14_Zn_5_As_12_O, although it is noticeably larger than those derived from Seebeck coefficient measurement or calculated using a simplified model with the LMTO code. A detailed study of thermoelectric properties (electrical resistivity, Seebeck, and thermal conductivity) via computational tools is anticipated to fully elucidate the thermoelectric efficiency of Eu_14_Zn_5_As_12_O and reconcile observed inconsistencies.

A static magnetic field of 13 kOe was applied to the sample of Eu_14_Zn_5_As_12_O to further probe the thermoelectric properties. The crystal was subjected to Hall effect measurements, which resulted in the Hall carrier concentration:
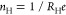
4where *e* is the electric charge, and *R*_H_ is the Hall coefficient.

An increase in charge carrier concentration, *n*, with temperature supports the semiconducting trend of the resistivity plot (Figure 6d). The *n* values range from ca. 2.8 × 10^16^ cm^–3^ at 300 K to ca. 8.0 × 10^17^ cm^–3^ at 500 K in the studied temperature region. A further increase in the electrical conductivity and carrier concentration is expected at elevated temperatures, although achieving the optimal carrier concentration of 10^19^ cm^–3^ order, typical for efficient thermoelectric materials, remains unlikely without doping. Undoped 1D Zintl pnictides, such as Ca_3_AlSb_3_, Sr_3_GaSb_3_, and Sr_3_ZnAs_3_, to name a few, often exhibit low carrier concentration, yet their thermoelectric performance is markedly improved through appropriate doping.^47,95,96^

The mobility of the primary charge carriers, identified as holes in Eu_14_Zn_5_As_12_O, linearly increases from approximately 4.1 to 6.4 cm^2^/Vs upon heating within the studied temperature range. These values are typical for Zintl phases featuring 1D polyanionic substructures, similar to Ca_3_AlSb_3_, Sr_3_AlSb_3_, and Sr_5_Al_2_Sb_6_.^91,95,97^ The mobility and overall thermoelectric performance of Eu_14_Zn_5_As_12_O may be significantly improved through strategic chemical doping. As discussed in Section 3.2.2., doping with Mn and/or Al is predicted to transform the anionic substructure into a 2D polyanionic network, potentially enhancing the thermoelectric properties of the proposed multinary phase.

### 3.5 Magnetic Properties

**Figure 7:**
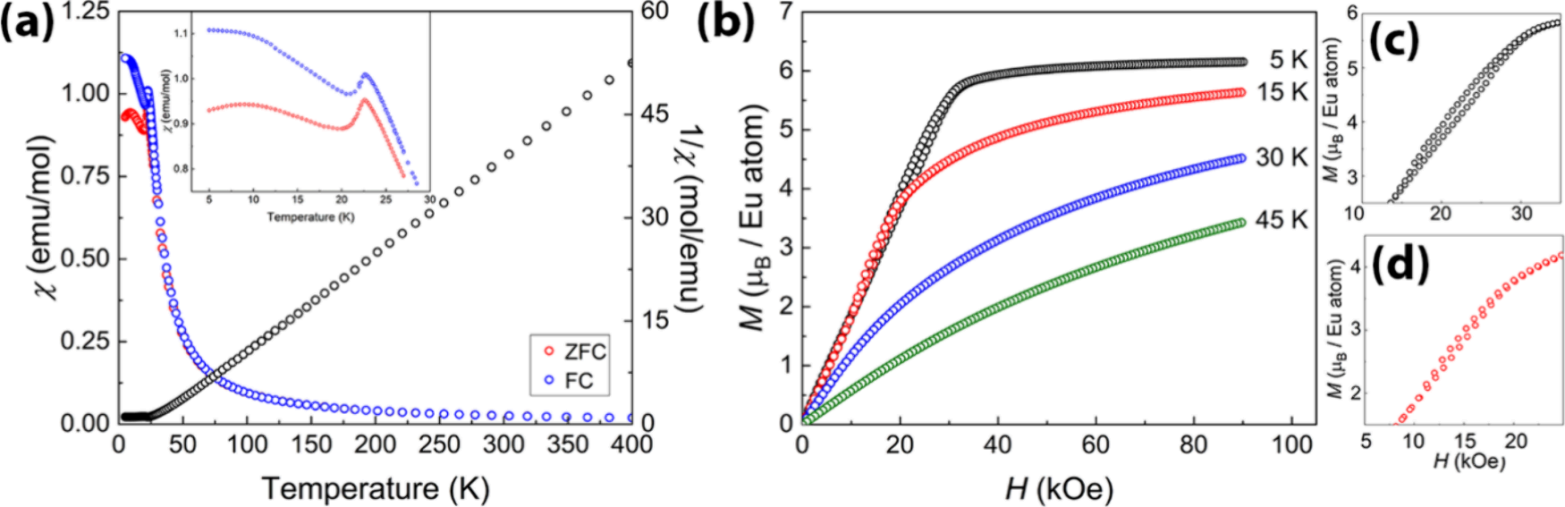
Magnetic properties of Eu_14_Zn_5_As_12_O. (a) Temperature dependence of the magnetic susceptibility (χ and χ^–1^ data) measured in zero field cooled (ZFC) and field cooled (FC) mode. (b) Magnetization isotherms measured at 5 K, 15 K, 30 K, and 45 K with detailed views of the hysteresis loops for the 5-K (c) and 15-K (d) data.

The temperature dependence of the magnetic susceptibility and the magnetic isotherms at *T* = 5 K, 15 K, 30 K, and 45 K for Eu_14_Zn_5_As_12_O are shown in Figure 7. The Curie–Weiss law is followed at the high-temperature part of the magnetic susceptibility, which allows for the linear fit of the inversed susceptibility data using the equation:
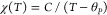
5where *C* is the Curie constant (*C* = *N*_A_*μ*_eff_^2^/3*k*_B_, *N*_A_ is Avogadro’s number, *μ*_eff_ is the effective magnetic moment) and *θ*_*P*_ is the paramagnetic Weiss temperature. The deduced magnetic moments are nearly identical for ZFC and FC modes (*μ*_eff_ = 7.53(2) μ_B_). This value is slightly lower than the theoretical value for the divalent Eu *μ*_calc_(Eu^2+^) = 7.94 *μ*_B_, which may be attributed to the residual Pb flux on the crystal surface or the presence of trivalent Eu^3+^ species. If the moment reduction is viewed as the presence of mixed Eu^2+^/Eu^3+^, ca. 9% of Eu would be in the Eu^3+^ state. This value is consistent with the fraction of Eu8 atoms (7.1%) that form short Eu8–O bonds (Figure 3e). However, further studies are needed to confirm the presence of a mixed magnetic state.

The Weiss constants, determined from the ZFC and FC fits, were consistent and positive (*θ_P_* = 25.5 K), being in line with the complex magnetic behavior observed at lower temperatures (Figure 7a). Eu_14_Zn_5_As_12_O shows two magnetic transitions at *T*_1_ = 22.6(1) K and *T*_2_ = 9.0(1) K, as indicated by the ZFC mode data. Both transitions can be assigned as antiferromagnetic based on the characteristic peak behavior. However, the FC mode measurements point to the antiferromagnetic nature of the first transition at *T*_*N*,1_ = 22.6(1) K, whereas a ferromagnetic nature to the lower-temperature transition is suggested, due to some coercivity evident below ∼ 7 K between the ZFC and FC curves. Data taken at lower temperatures, as well as heat capacity measurements, could help further elucidate the observed magnetic behavior. Such complex magnetic behavior is typical for Eu-bearing Zintl pnictides and was observed in such compounds as Eu_5_In_2_As_6_,^75^ Eu_5_In_2_Sb_6_,^88^ and Eu_3_InAs_3_.^98^

Measured magnetization isotherms (Figure 7b recorded above the magnetic phase transition (30 and 45 K) tend to linearize, as expected for a paramagnetic material. The 15-K isotherm below *T*_*N*,1_, but above the second broader transition displays a curved behavior, indicative of field-dependent spin orientation. The 5-K isotherm below both transitions shows a nearly linear increase followed by saturation (*H*_crit_ ≈ 35 kOe) with increasing field. We can also observe hysteresis loops for both the 5-K (Figure 7c) and 15-K data (Figure 7d), although it is less pronounced for the latter, indicating a ferromagnetic component to the magnetism at low temperatures. At *T* = 5 K and *H* = 90 kOe, the magnetization approaches 6.2(1) μ_B_, which is below the theoretical saturation moment of *μ*_eff_ = 7 *μ*_B_, as can be estimated from the *g*_*J*_ × *J* value.

A detailed examination of the crystal structure highlights the presence of multiple Eu crystallographic sites in Eu_14_Zn_5_As_12_O, but the Eu8 position requires special attention. As we discussed in Section 3.2.2, it is octahedrally coordinated by four As atoms and two O atoms (Figure S3) with [Eu8As_4_] planes aligning perpendicularly to the −Eu8–O–Eu8– linear chains. However, the Eu8 site can also be seen at the center of an almost perfect cube formed by Eu3 and Eu6 atoms (Figure 3e). The refined interatomic distances for Eu8–Eu6 and Eu8–Eu3 bonds are ca. 3.50 Å and 3.53 Å, respectively, being significantly shorter than the expected Eu–Eu distance based on the sum of covalent radii.^71^ We believe that the observed complex behavior may be significantly impacted by the presence of such short interatomic contacts between two magnetic elements, though further characterization is required to fully understand the magnetic structure of Eu_14_Zn_5_As_12_O.

## 4 Conclusions

We advanced the landscape of Zintl chemistry by introducing two quaternary heteroanionic arsenide oxides, Eu_8_Zn_2_As_6_O and Eu_14_Zn_5_As_12_O, which have been discovered and structurally characterized using single-crystal X-ray diffraction methods. They crystallize in novel structure types and feature unique, albeit heavily disordered, anionic substructures based on the corner-sharing arrangement of planar [ZnAs_3_] units. The uniqueness of crystal chemistry is attributed to two factors: (i) the versatility of Zn atoms to adopt trigonal-planar and tetragonal coordination and (ii) the diversification of compositions and structures through heteroanionicity, facilitated by the incorporation of varied anionic species. Electronic structure calculations and transport property measurements align well with the predicted charge-balanced semiconducting nature of (Eu^2+^)_8_(Zn^2+^)_2_(As^3–^)_6_(O^2–^) and (Eu^2+^)_14_(Zn^2+^)_5_(As^3–^)_12_(O^2–^), as envisioned by the Zintl–Klemm rules. The necessity for partial occupancy of Zn sites, essential for achieving the ideal charge balance, was confirmed through SCXRD analysis. Excellent ambient stability, high values of thermopower (*S*_500K_ = 220 μV K^–1^) and charge carrier concentration (*n*_500K_ = 8.0 × 10^17^ cm^–3^) underscore the significant potential for further enhancing the thermoelectric performance of Eu_14_Zn_5_As_12_O. Magnetic studies at low temperatures validate the presence of Eu^2+^, with an effective magnetic moment of *μ*_eff_ = 7.53(2) *μ*_B_, and reveal complex magnetic ordering characterized by two transition temperatures, partially due to noticeably shortened Eu–Eu contacts.

Expanding on the above, the thermoelectric performance of Eu_14_Zn_5_As_12_O could be optimized through targeted iso- and aliovalent doping with Mn^2+^, Cd^2+^, or Al^3+^ cations with preferred tetrahedral coordination. We anticipate increasing the dimensionality by introducing a 2D framework of corner-sharing tetrahedra, which could improve mobility and charge-carrier concentration to optimal levels. The further enhancement of complexity may result in the formation of other compounds within ternary systems like Eu–Al–As or Eu–Mn–As; therefore, a comprehensive understanding of the formation mechanisms and energies within these families of compounds is required. Detailed computational studies would also benefit the limits of thermoelectric efficiency for Eu_8_Zn_2_As_6_O and Eu_14_Zn_5_As_12_O, paving the way for the development of novel heteroanionic materials with optimized properties.

## Supporting Information Available

SEM images and EDX data; fractional atomic coordinates of Eu_8_Zn_2_As_6_O and Eu_14_Zn_5_As_12_O; selected interatomic distances in Eu_8_Zn_2_As_6_O and Eu_14_Zn_5_As_12_O; local coordination environments of Eu^2+^ cations in Eu_8_Zn_2_As_6_O and Eu_14_Zn_5_As_12_O; representation of the structural models used for electronic structure calculations, PDOS and selected COHP curves for selected bonds in Eu_8_Zn_2_As_6_O and Eu_14_Zn_5_As_12_O (PDF)
