# Supplementary material for: Advancing Heteroanionicity in Zintl Phases: Crystal Structures, Thermoelectric and Magnetic Properties of Two Quaternary Semiconducting Arsenide Oxides, Eu8Zn2As6O and Eu14Zn5As12O
Source: Inorg Chem. 2024 Jun 21;63(43):20226–39. doi: 10.1021/acs.inorgchem.4c01580 (PMC11523219; doi:10.1021/acs.inorgchem.4c01580)
Supplement: Supplementary file 1 — ic4c01580_si_001.pdf [file ic4c01580_si_001.pdf]

Supporting Information

Advancing Heteroanionicity in Zintl Phases: Crystal Structures, Thermoelectric and Magnetic Properties of Two Quaternary Semiconducting Arsenide Oxides, $Eu_8Zn_2As_6O$ and $Eu_{14}Zn_5As_{12}O$

Mohd Ishtiyak,[1,#] Spencer R. Watts,[1,#] Bhushan Thipe,[2] Frank Womack,[2] Philip Adams,[2] Xiaojian Bai,[2] David P. Young,[2] Svilen Bobev,[3] Sviatoslav Baranets[1,]*

[1] Department of Chemistry, Louisiana State University, Baton Rouge, Louisiana, 70803, United States

[2] Department of Physics & Astronomy, Louisiana State University, Baton Rouge, Louisiana, 70803, United States

[3] Department of Chemistry and Biochemistry, University of Delaware, Newark, Delaware 19716, United States

[#] These authors contributed equally

* Corresponding author, email: sbaranets@lsu.edu

Table of Contents

1. Figure S1. EDX data and SEM images collected on single crystals of $Eu_8Zn_2As_6O$ and $Eu_{14}Zn_5As_{12}O$ S3

2. Table S1. Fractional atomic coordinates and equivalent displacement parameters for $Eu_8Zn_2As_6O$ S4

3. Table S2. Fractional atomic coordinates and equivalent displacement parameters for $Eu_{14}Zn_5As_{12}O$ S5

4. Table S3. Selected interatomic distances in $Eu_8Zn_2As_6O$ and $Eu_{14}Zn_5As_{12}O$ S6

5. Figure S2. Local coordination environment of Eu^{2+} cations in the crystal structure of $Eu_8Zn_2As_6O$ S7

6. Figure S3. Local coordination environment of Eu^{2+} cations in the crystal structure of $Eu_{14}Zn_5As_{12}O$ S7

7. Figure S4. Structural model of the anionic substructure of $Eu_8Zn_2As_6O$ used for electronic structure calculations S8

8. Figure S5. A unit cell of the $Eu_{14}Zn_6As_{12}O$ model devoid of the disorder used for electronic structure calculations S8

9. Figure S6. PDOS plots of $Eu_8Zn_2As_6O$ and $Eu_{14}Zn_5As_{12}O$ S9

10. Figure S7. COHP curves for selected bonds in $Eu_8Zn_2As_6O$ and $Eu_{14}Zn_5As_{12}O$ S10

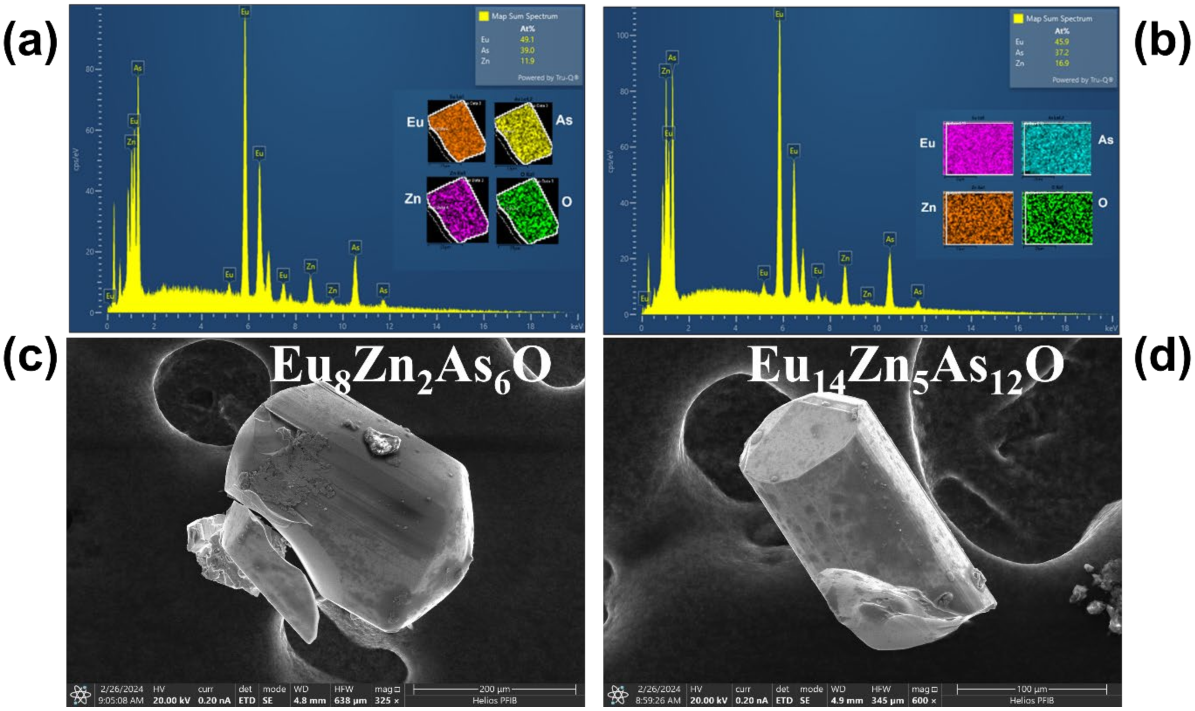

Figure S1. EDX data and SEM images collected on single crystals of $Eu_8Zn_2As_6O$ **(a)** and $Eu_{14}Zn_5As_{12}O$ **(b)**. Inset colored pictures in (a) and (b) exhibit the elemental mapping. Chemical composition is provided in at. %. Theoretical values with excluded oxygen are $w_{at.}$(Eu) = 50.0%, $w_{at.}$(Zn) = 12.5%, $w_{at.}$(As) = 37.5% for $Eu_8Zn_2As_6O$ and $w_{at.}$(Eu) = 45.2%, $w_{at.}$(Zn) = 16.1%, $w_{at.}$(As) = 38.7% for $Eu_{14}Zn_5As_{12}O$. (c) and (d) exhibit the representative SEM images of the crystals of $Eu_8Zn_2As_6O$ and $Eu_{14}Zn_5As_{12}O$, respectively.

Table S1. Fractional atomic coordinates and equivalent anisotropic displacement parameters U_{eq} values for $Eu_8Zn_2As_6O$.

Atoms	Wyckoff Site	*x*	*y*	*z*	U_{eq}[a] (Å^2)
Eu1A[b]	8*c*	0.1203(9)	0.2224(5)	0.1179(3)	0.005(1)
Eu1B[b]	8*c*	0.1251(2)	0.2103(3)	0.1135(1)	0.005(1)
Eu2A[b]	8*c*	0.1348(13)	0.0115(4)	0.1591(3)	0.007(1)
Eu2B[b]	8*c*	0.1088(6)	0.0168(2)	0.1607(1)	0.007(1)
Eu3A[b]	8*c*	0.1308(7)	0.4113(3)	0.0284(3)	0.005(1)
Eu3B[b]	8*c*	0.1241(2)	0.4132(1)	0.0184(2)	0.005(1)
Eu4A[b]	8*c*	0.1400(4)	0.0183(1)	0.3398(1)	0.008(1)
Eu4B[b]	8*c*	0.1045(17)	0.0139(4)	0.3413(3)	0.008(1)
Eu5	8*c*	0.1241(1)	0.4110(1)	0.4734(1)	0.005(1)
Eu6	8*c*	0.1257(1)	0.2216(1)	0.3829(1)	0.006(1)
Eu7	8*c*	0.3750(1)	0.2494(1)	0.0005(1)	0.004(1)
Eu8	8*c*	0.3748(1)	0.3199(1)	0.2504(1)	0.008(1)
Zn1A[b]	8*c*	0.375(2)	0.1103(7)	0.2766(9)	0.016(1)
Zn1B[b]	8*c*	0.3752(2)	0.1099(1)	0.2418(1)	0.016(1)
Zn1C[b]	8*c*	0.3740(8)	0.1150(3)	0.2170(4)	0.016(1)
Zn2[b]	8*c*	0.1245(2)	0.3923(1)	0.1554(1)	0.006(1)
Zn3[b]	8*c*	0.1252(11)	0.3928(4)	0.3452(3)	0.022(2)
As1	8*c*	0.1248(1)	0.1792(1)	0.2493(1)	0.009(1)
As2	8*c*	0.1249(1)	0.4611(1)	0.2490(1)	0.005(1)
As3	8*c*	0.3648(1)	0.3461(1)	0.1132(1)	0.006(1)
As4	8*c*	0.3749(1)	0.0887(1)	0.0770(1)	0.007(1)
As5	8*c*	0.3751(1)	0.0904(1)	0.4261(1)	0.005(1)
As6	8*c*	0.3850(1)	0.3474(1)	0.3876(1)	0.006(1)
O1	8*c*	0.1241(9)	0.2568(3)	0.4940(2)	0.018(1)

[a] *Ueq* is defined as 1/3 of the trace of the orthogonalized *Uij* tensor.
[b] Atomic occupancies according to the refinement: 1 = 0.20(3)Eu1A + 0.80Eu1B; 1 = 0.21(3)Eu2A + 0.79Eu2B; 1 = 0.24(2)Eu3A + 0.76Eu3B; 1 = 0.875(14)Eu4A + 0.125Eu4B; 1 = 0.059(3)Zn1A + 0.764(6)Zn1B + 0.176(5)Zn1C; Zn2 = 0.75 (constrained), Zn3 = 0.132(4).

Table S2. Fractional atomic coordinates and equivalent anisotropic displacement parameters U_{eq} values for $Eu_{14}Zn_5As_{12}O$.

Atoms	Wyckoff Site	*x*	*y*	*z*	U_{eq}[a] (Å^2)
Eu1A[b]	2*m*	0.3299(3)	0	0.3174(1)	0.006(1)
Eu1B[b]	2*m*	0.3538(3)	0	0.3255(2)	0.006(1)
Eu2A[b]	2*m*	0.5285(4)	0	0.1265(1)	0.016(1)
Eu2B[b]	2*m*	0.5579(7)	0	0.1351(3)	0.007(1)
Eu3	2*n*	0.0716(1)	1/2	0.1672(1)	0.007(1)
Eu4	2*n*	0.1934(1)	1/2	0.7256(1)	0.006(1)
Eu5[b]	4o	0.3306(1)	0.4709(7)	0.5278(1)	0.009(1)
Eu6	2*n*	0.7686(1)	1/2	0.0233(1)	0.010(1)
Eu7	1*f*	0	1/2	½	0.004(1)
Eu8	1*a*	0	0	0	0.006(1)
Zn1A[b]	2*m*	0.0147(1)	0	0.3261(1)	0.008(1)
Zn1B[b]	2*m*	0.0351(3)	0	0.3539(3)	0.008(1)
Zn2A[b]	2*m*	0.6109(1)	0	0.3450(1)	0.009(1)
Zn2B[b]	2*m*	0.6282(4)	0	0.3205(3)	0.009(1)
Zn3A	2*n*	0.3668(2)	1/2	0.1502(1)	0.011(1)
Zn3B	2*n*	0.3472(5)	1/2	0.1734(3)	0.011(1)
As1	2*m*	0.1350(1)	0	0.8451(1)	0.008(1)
As2	2*m*	0.1631(1)	0	0.5930(1)	0.004(1)
As3	2*m*	0.2470(1)	0	0.1226(1)	0.008(1)
As4	2*n*	0.1404(1)	1/2	0.3520(1)	0.004(1)
As5	2*n*	0.5311(1)	1/2	0.2740(1)	0.006(1)
As6[b]	2*m*	0.5081(1)	0	0.4848(1)	0.004(1)
As7[b]	2*n*	0.4914(2)	1/2	0.0135(1)	0.005(1)
O	1*b*	0	1/2	0	0.016(1)

[a] *Ueq* is defined as 1/3 of the trace of the orthogonalized *Uij* tensor.

[b] Atomic occupancies according to the refinement: 1 = 0.701(12) Eu1A + 0.299Eu1B; 1 = 0.676(17)Eu2A + 0.324Eu2B; 1 = 0.707(5)Zn1A + 0.293Zn1B; 0.75 = 0.549(2)Zn2A + 0.201Zn2B; 0.75 = 0.545(2)Zn3A + 0.205Zn3B; SOFs of Eu5, As6, and As7 were constrained to 0.5.

Table S3. Selected interatomic distances in $Eu_8Zn_2As_6O$ and $Eu_{14}Zn_5As_{12}O$.

Atom pair	Distance (Å)	Atom pair	Distance (Å)	Atom pair	Distance (Å)	Atom pair	Distance (Å)
			**$Eu_8Zn_2As_6O$**				
Eu1A–O1	2.859(8)	Eu1A–As4	3.358(9)	Eu3A–As5	3.218(6)	Eu5–As6	3.249(1)
Eu1B–O1	2.792(5)	Eu1A–As5	3.302(8)	Eu3A–As6	3.127(6)	Eu6–As1	3.1415(6)
Eu3A–O1	2.951(8)	Eu1A–As6	3.00(9)	Eu3B–As3	3.274(5)	Eu6–As3	3.162(1)
Eu3B–O1	2.929(5)	Eu1B–As1	3.154(2)	Eu3B–As4	3.259(2)	Eu6–As4	3.320(1)
Eu5–O1	2.651(5)	Eu1B–As3	3.158(3)	Eu3B–As4	3.136(5)	Eu6–As5	3.313(1)
Eu6–O1	2.614(5)	Eu1B–As4	3.164(5)	Eu3B–As5	3.098(3)	Eu6–As6	3.1659(1)
Eu7–O1 × 2	2.257(8)	Eu1B–As5	3.167(3)	Eu3B–As6	3.244(3)	Eu7–As3	3.0563(6)
Zn1A–As1 × 2	2.61(2)	Eu1B–As6	3.176(4)	Eu4A–As1	3.425(1)	Eu7–As4	3.2330(6)
Zn1A –As2	2.60(1)	Eu2A–As1	3.510(7)	Eu4A–As2	3.286(3)	Eu7–As5	3.1994(6)
Zn1B–As1	2.549(2)	Eu2A–As2	3.264(9)	Eu4A–As2	3.125(3)	Eu7–As6	3.0611(6)
Zn1B–As1	2.554(2)	Eu2A–As2	3.111(9)	Eu4A–As4	3.283(3)	Eu8–As1	3.280(1)
Zn1B –As2	2.520(1)	Eu2A–As3	2.988(7)	Eu4A–As5	3.147(3)	Eu8–As1	3.279(1)
Zn1C–As2	2.627(7)	Eu2A–As4	3.154(9)	Eu4A–As6	3.098(2)	Eu8–As2	3.288(1)
Zn1C –As1	2.606(7)	Eu2A–As5	3.331(9)	Eu4B–As1	3.505(7)	Eu8–As2	3.287(1)
Zn1C –As1	2.704 (6)	Eu2B–As1	3.419(3)	Eu4B–As2	3.35(1)	Eu8–As3	3.1757(6)
Zn2–As2	2.4402(9)	Eu2B–As2	3.101(4)	Eu4B–As2	3.06(1)	Eu8–As6	3.1793(6)
Zn2–As3	2.501(1)	Eu2B–As2	3.281(4)	Eu4B–As4	3.07(1)	Eu1A–Zn2	3.000(9)
Zn2 –As6	2.495(1)	Eu2B–As3	3.093(3)	Eu4B–As5	3.38(1)	Eu1A–Eu7	3.534(7)
Zn3–As2	2.487(6)	Eu2B–As4	3.306(4)	Eu4B–As6	3.011(7)	Eu1B–Eu2A	3.521(8)
Zn3–As3	2.657(9)	Eu2B–As5	3.156(4)	Eu5–As3	3.258(1)	Eu3A–Eu7	3.572(6)
Zn3–As6	2.653(9)	Eu3A–As3	3.073(7)	Eu5–As4	3.280(1)	Eu3B–Eu7	3.580(2)
Eu1A–As1	3.097(6)	Eu3A–As4	3.201(6)	Eu5–As5	3.217(1)		
Eu1A–As3	3.045(8)	Eu3A–As4	3.341(7)	Eu5–As5	3.2209(6)		
			**$Eu_{14}Zn_5As_{12}O$**				
Eu6–O	2.7026(3)	Zn3B–As3 × 2	2.564(3)	Eu2A–As1	3.403(8)	Eu5–As6	3.072(2)
Eu8–O × 2	2.2274(1)	Zn3B–As5	2.394(5)	Eu2A–As5 × 2	3.262(2)	Eu5–As6	3.254(2)
Eu3–O	2.7379(3)	Eu1A–As3	3.189(2)	Eu2A–As7 × 2	3.291(4)	Eu6–As1 × 2	3.1609(4)
Zn1A–As1	3.012(2)	Eu1A–As4 × 2	3.209(2)	Eu2A–As7 × 2	3.002(4)	Eu6–As3 × 2	3.2724(4)
Zn1A–As2	2.613(1)	Eu1A–As5 × 2	3.350(2)	Eu3–As1 × 2	3.1963(4)	Eu6–As7	3.088(2)
Zn1A –As4 × 2	2.6281(9)	Eu1A–As6	3.096(4)	Eu3–As3 × 2	3.1545(4)	Eu6–As7	2.867(2)
Zn1B–As2	2.549(3)	Eu1A–As6	3.434(3)	Eu3–As4	3.0165(5)	Eu7–As2 × 4	3.1017(3)
Zn1B–As4 × 2	2.525(2)	Eu1B–As3	3.350(4)	Eu4–As1 × 2	3.1351(4)	Eu7–As4 × 2	3.1658(5)
Zn2A–As2	2.549(2)	Eu1B–As4 × 2	3.363(3)	Eu4–As2 × 2	3.1040(4)	Eu8–As1 × 2	3.2277(5)
Zn2A–As5 × 2	2.598(1)	Eu1B–As5 × 2	3.212 (3)	Eu4–As5	3.0947(6)	Eu8–As3 × 2	3.1104(5)
Zn2A–As6	2.785(2)	Eu1B–As6	3.233(4)	Eu5–As2	3.147(2)	Eu1A–Zn2A	3.102(4)
Zn2B–As2	2.503(5)	Eu1B–As6	2.871(4)	Eu5–As2	3.325(2)	Eu1B–Zn2A	2.845(4)
Zn2B–As5 × 2	2.534(2)	Eu2A–As3	3.152 (4)	Eu5–As4	3.2724(6)	Eu2A–Zn3A	2.948(3)
Zn3A–As3 × 2	2.5973(9)	Eu2A–As5 × 2	3.307(1)	Eu5–As5	3.3601(6)	Eu6–Eu8	3.5022(2)
Zn3A–As5	2.485(2)	Eu2A–As7 × 2	2.890(1)	Eu5–As6	3.005(2)		
Zn3A–As7	2.886(2)	Eu2A–As7 × 2	3.197(1)	Eu5–As6	2.807(2)		

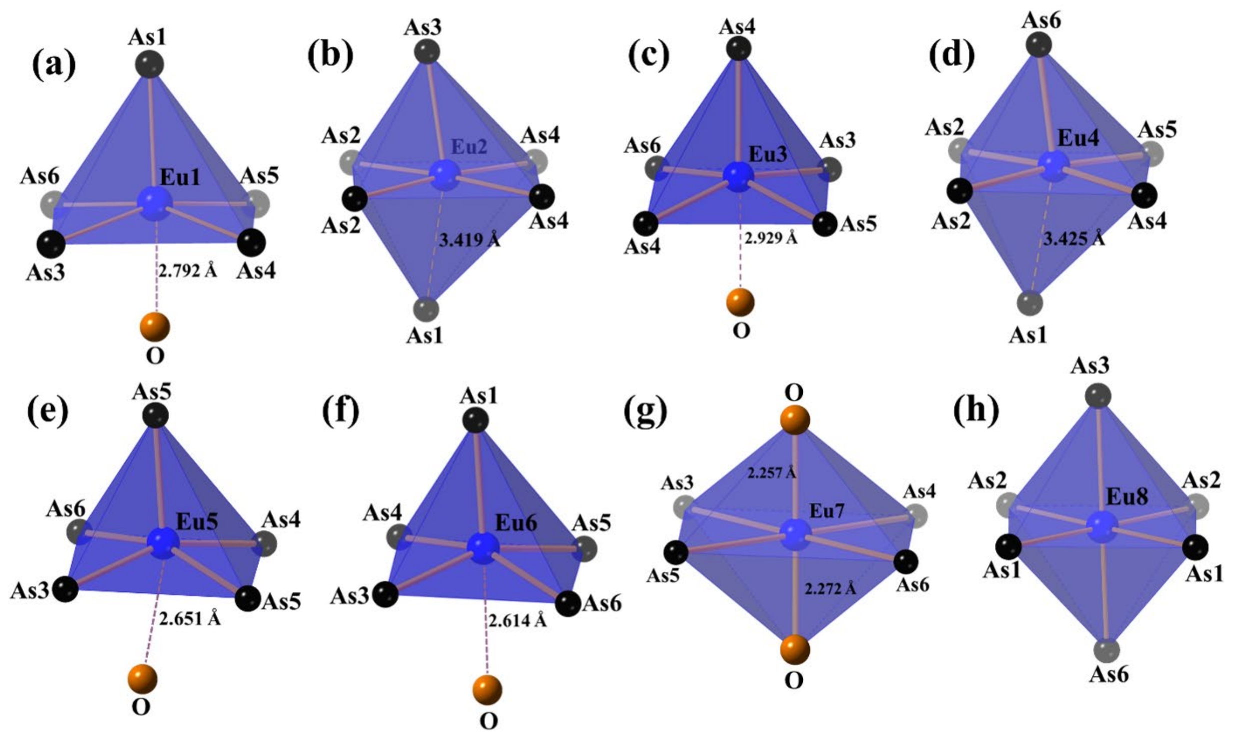

Figure S2. (a)-(h) Local coordination environment of Eu^{2+} cations in the crystal structure of $Eu_8Zn_2As_6O$. Eu atoms are blue, As atoms are black and grey, O atoms are orange.

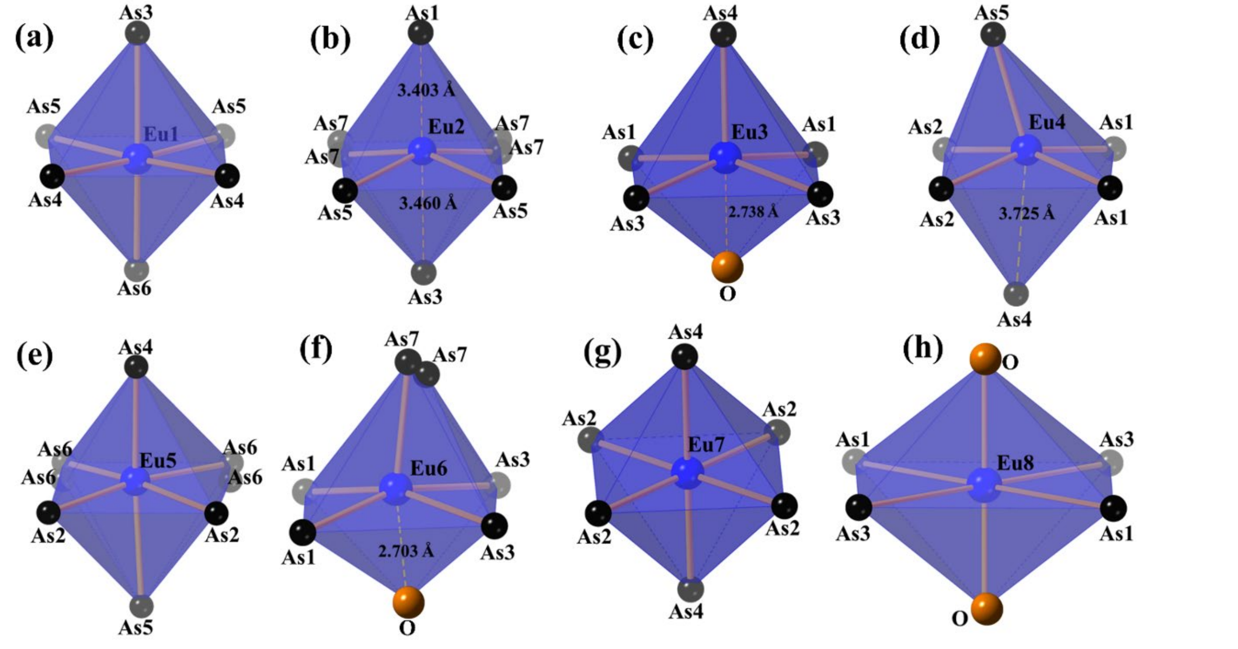

Figure S3. (a)-(h) Local coordination environment of Eu^{2+} cations in the crystal structure of $Eu_{14}Zn_5As_{12}O$. Eu atoms are blue, As atoms are black and grey, O atoms are orange.

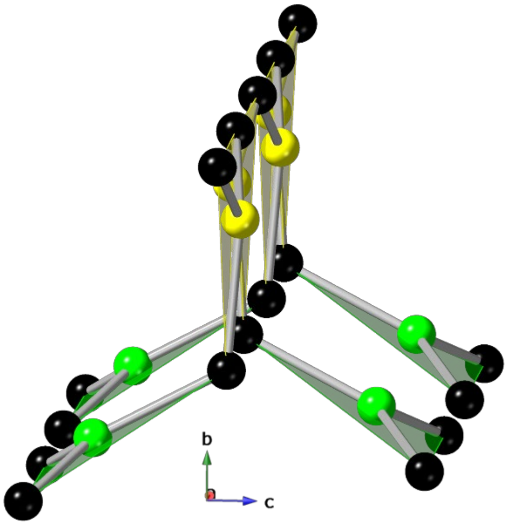

Figure S4. Structural model of the anionic substructure of $Eu_8Zn_2As_6O$ used for electronic structure calculations. Here, Zn1 and Zn2 atoms are shown in yellow and green colors while As atoms are depicted in black.

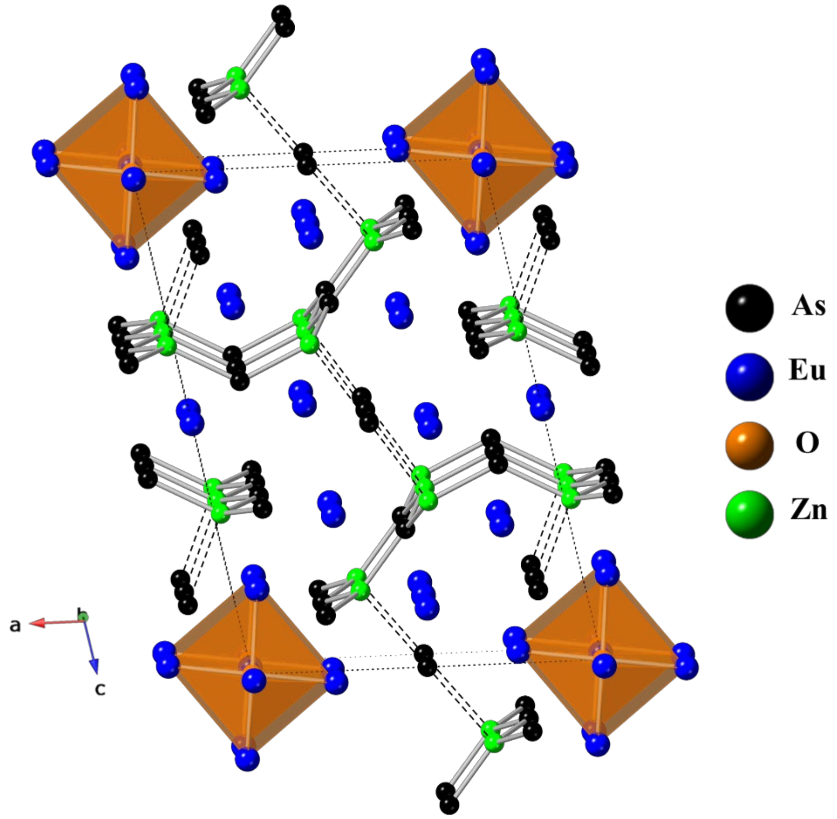

Figure S5. A unit cell of $Eu_{14}Zn_6As_{12}O$ devoid of the disorder. This simplified 2-electron-rich structural model was used for the electronic structure calculations. Dashed bonds denote unrealistic Zn–As interatomic distances above 3 Å.

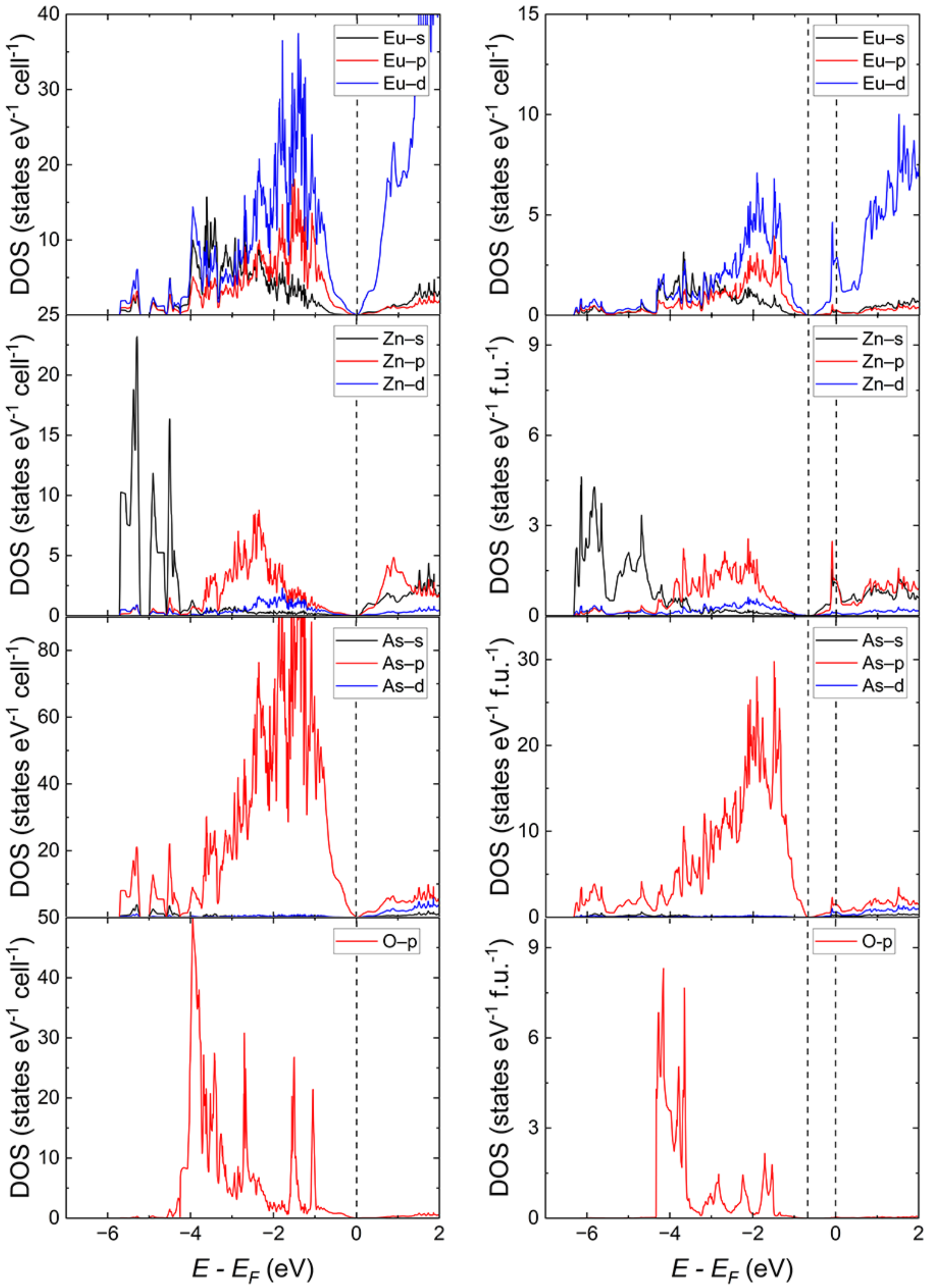

Figure S6. Calculated partial density of states (PDOS) plots for $Eu_8Zn_2As_6O$ (left) and $Eu_{14}Zn_6As_{12}O$ (right). The Fermi level is the energy reference at 0 eV. An additional dashed line at ca. –0.69 eV indicates a 2-election shift corresponding to the $Eu_{14}Zn_5As_{12}O$ composition.

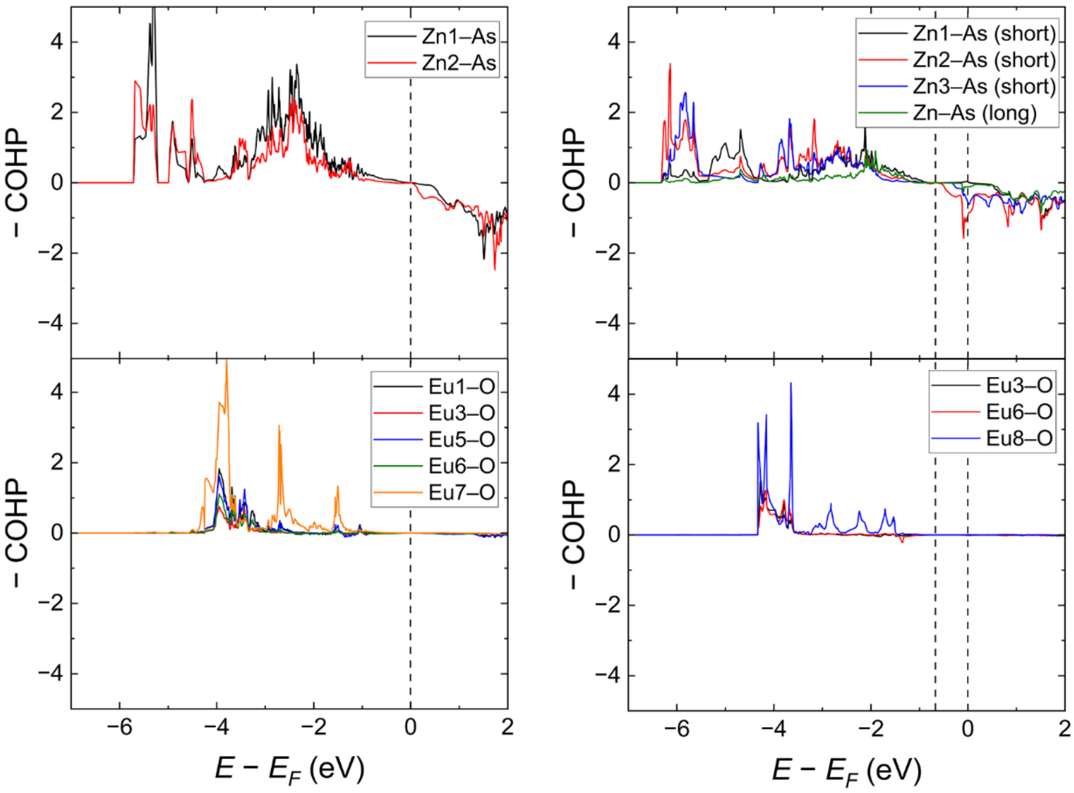

Figure S7. COHP plots for selected interatomic contacts observed in $Eu_8Zn_2As_6O$ (left) and $Eu_{14}Zn_6As_{12}O$ (right). The Fermi level is the energy reference at 0 eV. An additional dashed line at ca. –0.69 eV indicates a 2-election shift corresponding to the $Eu_{14}Zn_5As_{12}O$ composition.
